# Antibacterial and physicomechanical properties of cellulosic nonwovens functionalized with chitosan: a study on interaction effects of influencing factors and assessment methods

**DOI:** 10.1186/s40643-025-00843-2

**Published:** 2025-02-15

**Authors:** Esubalew Kasaw Gebeyehu, Rekha Shresth, Tonmoy Saha, Jenni Tienaho, Ulla Jauhiainen, Ali Amin Tarhini, Ali Reza Tehrani-Bagha

**Affiliations:** 1 Department of Bioproducts and Biosystems, School of Chemical Engineering, Aalto University, Vuorimiehentie 1, 02150 Espoo, Finland; 2 Natural Resources Institute Finland (Luke), Latokartanonkaari 9, 00790 Helsinki, Finland

**Keywords:** Cellulosic nonwoven, Chitosan, Antibacterial activity, Absorption and diffusion test, Culture medium dynamics, Physio-mechanical properties

## Abstract

The growing demand for antimicrobial textiles and environmental concerns over synthetic agents have driven interest in biobased agents like chitosan, an eco-friendly alternative reported with variable effectiveness on textiles. This research investigates the effects of chitosan’s molecular weight, concentration, treatment method, and their interaction effects on the antibacterial and physicomechanical properties of cellulosic nonwovens. The role of culture medium dynamics in antibacterial testing was also examined. Chitosan with low (30 kDa), medium (250 kDa) and high (2100 kDa) molecular weights at concentrations of 1 to 15 g/L was applied using pad-dry and dip-dry methods, with dip coating resulting in higher chitosan deposition. Antibacterial activity against *Escherichia coli (E. coli)* was assessed using three methods: agar diffusion, a luminescent bacterial biosensor assay, and log reduction of colony-forming units (CFU/mL) in a time-kill test. While the first two test methods showed no antibacterial effect, CFU/mL revealed significant activity with interaction effects showing that the dip-coated, 30 kDa chitosan above a concentration of 5 g/L achieved complete bacterial reduction. The enhanced antibacterial performance of chitosan-coated cellulosic substrates, compared to chitosan in a culture medium, highlighted the role of cellulosic nonwoven in boosting antibacterial effectiveness, likely through improved contact and interaction with bacterial cells. The study demonstrated that chitosan-treated cellulosic nonwovens, particularly with dip-dry coating altered tensile strength and increased the bending resistance and bending stiffness.

**Graphical Abstract:**

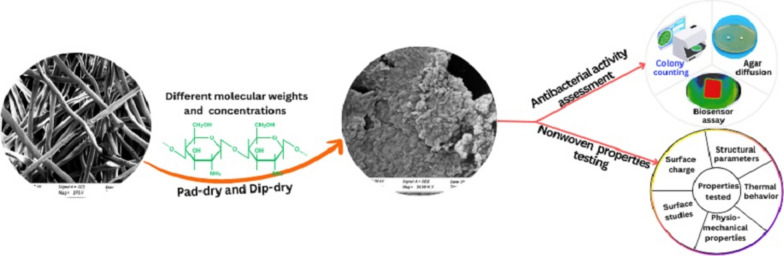

**Supplementary Information:**

The online version contains supplementary material available at 10.1186/s40643-025-00843-2.

## Introduction

Textiles are widely used to meet essential human needs in clothing and various technical applications. Textiles from natural sources generally possess attributes like biodegradability and renewability. Among these, cellulosic fibers due to their large surface area, suitable carbon content and ability to retain moisture make them conducive to microbial growth (Gulati et al. 2022). These microorganisms can rapidly multiply under favorable conditions such as moisture, nutrients and temperature leading to undesirable effects on both the textile itself and the user, Table 1 (Gupta 2007; Morais et al. 2016; Windler et al. 2013). This is particularly critical given the increased use of textiles in various sectors such as medical, construction, automotive, industrial, and agricultural and their continuous environmental exposure.

**Table 1:** Effect of harmful bacteria on textiles and humans (Gupta 2007)

Effect of bacteria	Impact on humans	Impact on textiles
Microbial proliferation	Causes unpleasant body odor due to bacterial activity	Accelerates fabric degradation, affecting texture and strength
Degradation	Increases risk of pathogenic infections and allergies	Results in discoloration, stains, and aesthetic deterioration
Hygiene compromise	Transfers harmful pathogens through contact	Requires frequent laundering to maintain hygiene and appearance

Cellulosic nonwovens used in healthcare environments are particularly required to possess antimicrobial properties to prevent the spread of pathogenic infections (Adamu et al. 2021; Morris and Murray 2020; Rogina-Car et al. 2020). Ensuring textiles remain free from bacterial contamination is essential to maintain their integrity, usability and safety (Gupta 2007).

Currently, there are different antibacterial agents used for textiles with their advantages and disadvantages. Synthetic antibacterial agents like metal and metal compounds (Xing et al. 2023) such as copper, zinc, cobalt, silver, and metal oxide nanoparticles are commonly used for textiles. Despite their effectiveness, these agents pose environmental and health risks due to their persistence and potential for bioaccumulation during synthesis and application (Nowack et al. 2011; Zhang et al. 2021). Triclosan, another widely used agent, has bactericidal and bacteriostatic effects but releases by-products like methyl-triclosan and chlorinated phenols during washing, which are more toxic and less degradable than the parent compound. This has led to increased regulatory restrictions (Dann and Hontela 2011; Uddin 2014). Similarly, quaternary ammonium compounds, formed from tertiary amine alkylation with halocarbons, are non-biodegradable and can cause allergic reactions upon skin contact (Warshaw et al. 2007), raising long-term environmental and health concerns (Gerba 2015).

A major concern with synthetic antibacterial agents is their lack of selectivity for pathogenic microorganisms. For example, while N-halamines and polybiguanides are effective and have less environmental impact, their application in daily-use products that come into direct contact with skin is limited. As the problem with most antibacterial agents, these agents can kill both pathogenic microorganisms and normal skin flora, which is essential for protecting against the colonization and growth of harmful germs. This poses a significant risk for products designed for regular skin contact (SanMiguel et al. 2017).

Regulatory frameworks such as European Oeko-Tex Standard 100 and biocidal productive 98/8/EEC directive (Commission 1998) focuses on limiting the use of harmful antimicrobial substances in textiles and encouraging safer alternatives. Hence, there is a global shift in exploring biobased antimicrobial agents nowadays. Natural antimicrobial agents such as biopolymers and plant extracts offer environmentally friendly alternatives (Adamu et al. 2022). However, their variable composition and potential for skin sensitization limit their widespread application. Additionally, these agents often require high concentrations to achieve the desired antimicrobial effect (Burt 2004) which can affect the physio-mechanical properties in the case of textile applications that require thorough optimization.

Chitosan is a natural cationic biopolymer derived from deacetylated chitin which presents a promising alternative due to its unique properties such as non-toxic, biodegradability, biocompatibility and antimicrobial activity against a broad spectrum of microorganisms, including bacteria, fungi and yeasts (Rabea et al. 2003). These characteristics make chitosan an attractive option for different applications including medical work uniforms, wound dressings (Li et al. 2023), bandages, sutures and filtration materials. However, studies on the antibacterial effectiveness of chitosan-treated textiles have shown inconsistent results. While some studies report highly effective antibacterial activity, others have found negligible effects (Fouilloux et al. 2024; Yasser et al. 2023) highlighting the need for further research to better understand its performance in different conditions.

Despite abundant research on the antimicrobial properties of chitosan, significant gaps remain:

(i) There is a need to understand how different application strategies influence its antibacterial activity, particularly on nonwoven fabrics. Different application methods can significantly affect the distribution and hence the antibacterial efficiency of chitosan on textile substrates (Roy et al. 2017). The traditional padding and dipping application methods have been shown to impart antimicrobial properties of chitosan onto fabrics, but their comparative effectiveness under uniform conditions remains under researched. (ii) The molecular weight and concentration of chitosan are critical factors influencing its antimicrobial efficiency. Studies (Liu et al. 2004; Zheng and Zhu 2003) have demonstrated that the antibacterial activity of chitosan films increased with its molecular weight and concentration due to its higher viscosity and film-forming capabilities. Despite these findings, there remains a lack of comprehensive studies that systematically compare the effects of different molecular weights and concentrations across various application methods. (iii) The impact of the incorporation of chitosan on the physio-mechanical properties of nonwoven textiles at different molecular weights and concentrations of chitosan remains unexplored. (iv) Different antibacterial assessment methods exist for leaching and non-leaching agents used on treated textiles (Pinho et al. 2011). The suitability and accuracy of these methods in evaluating the antibacterial efficiency of chitosan-treated nonwovens need a thorough examination. (v) Investigating whether the interaction between cellulose and chitosan enhances antibacterial properties could provide valuable insights for developing more effective antibacterial textiles. To examine this, similar concentrations of chitosan applied to cellulosic textiles were assessed for antibacterial properties in culture media. These results were compared with the antibacterial properties of equivalent concentrations of chitosan-functionalized nonwoven materials to evaluate their combined effectiveness. This aspect of the synergistic effect between cellulose and chitosan has not been previously explored, according to our information.

This study aims to evaluate the antibacterial efficiency of chitosan with low, medium, and high molecular weights at different concentrations. It compares the antibacterial effectiveness of padding and dip coating methods and examines their effect on the physicochemical properties of nonwoven substrates. Additionally, the antibacterial performance of treated samples was assessed against *E. coli* using three distinct methods: by diffusion method, luminescent bacterial biosensor method, and CFU/mL at 0 and 24 h (h). Furthermore, to understand how well chitosan can inhibit or kill *E. coli* in different environments antibacterial activity tests were conducted on chitosan both in culture medium and within cellulosic textiles. This analysis considered factors such as chitosan diffusion, possible synergistic effects and how the medium itself might influence these properties. The findings from this study will provide valuable insights for the optimization of the use of chitosan for enhanced antibacterial performance in nonwoven fabrics, with implications for the development of advanced antimicrobial textiles.

## Materials and methods

### Materials

#### A. Fabric

In this study, we used lyocell nonwoven with a basis weight of 58.2 g/m^2^ and a thickness of 265 ± 4.1 µm which was supplied by Suominen Oy—Finland.

#### B. Chemicals

The antimicrobial agent chitosan with a molecular weight of 30 kDa (LMW), 250 kDa (MMW), and 2100 kDa (HMW) was purchased from Sigma-Aldrich. Acetic acid and sodium acetate of analytical grade were used for aqueous acetic acid preparation. For microbiological analysis, Muller Hinton Agar (MHA) and Luria Bertani Agar (LB agar) were bought from Sigma-Aldrich.

#### C. Test microorganism

The test organism *Escherichia coli* (Migula) Castellani and Chalmers (*E. coli*), ATCC 25922 strain was purchased from ATCC: the global bioresource center. The genetically modified E. *coli* K12 + pcGLS11 was a kind gift from the authors of the paper (Vesterlund et al. 2004).

### Methods

#### A. Chitosan solution preparation

Chitosan solutions of 1, 5, 10, and 15 g/L concentrations were prepared for each LMW, MMW, and HMW chitosan by dissolving in acetic acid solution. To maintain the pH at 5, a small amount of sodium acetate was added, just enough to stabilize the pH without affecting the overall composition of the solution. The mixtures were continuously stirred with a magnetic stirrer at a speed of 1000 rpm until it gets completely dissolved.

#### B. Antimicrobial treatment of nonwoven

The two common fabric post-treatment methods were used to apply the chitosan solution to the nonwoven. A split-plot factorial design was implemented to evaluate the individual and interaction effects of variables. The application methods were treated as the whole-plot factor, while molecular weight and concentration were randomized as sub-plot factors.

In the Pad-dry (P × D) application method the nonwoven was dipped in the chitosan solution for two minutes and the fabric was passed through a one nip padding mangle under a constant pressure of 0.2 MPa and a speed of 15 m/min to squeeze excess liquor and ensure proper distribution and attachment of chitosan molecules onto nonwoven fabric. In the dip-dry (D × D) application, the samples were soaked in a chitosan solution for two minutes. After each treatment, the nonwoven fabric was dried at 100 °C in an oven for 5 min.

#### C. Performance evaluation

##### i. Antibacterial assessment

Each LB broth and MHA was mixed with distilled water and boiled with continuous stirring until fully dissolved and then autoclaved for further use. *E. coli* bacterial inoculum was rehydrated from its freeze-dried form and plated on LB agar media for antimicrobial testing based on ATCC 25922 recommendation. A single *E. coli* colony was picked and incubated in LB broth. The suspension was diluted to the desired concentration for further testing. For a comparative analysis of different test methods to verify the antimicrobial action of synergized chitosan on nonwoven is based on leachability or non-leachability, the antibacterial effectiveness of chitosan-treated samples was evaluated using three different test methods.

**Fig. 1:**
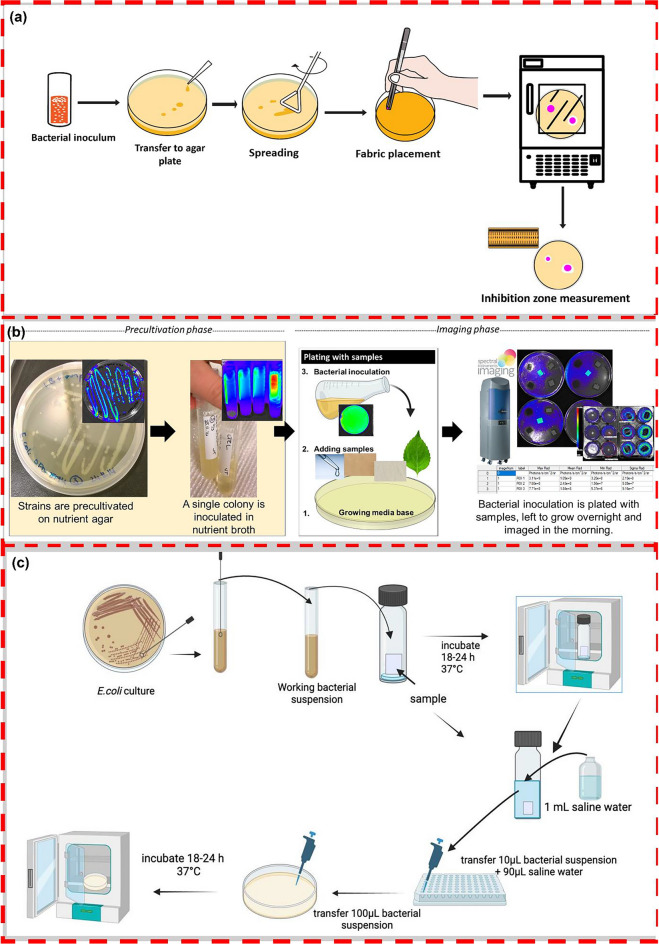
antibacterial assessment procedure for **a** agar diffusion test method using AATCC TM 147 **b** simplified workflow of the method with bioluminescent bacteria **c** colony counting test method

The first method was the AATCC TM147 qualitative agar diffusion test method. In this test procedure as shown in Fig. 1a, a colony from a culture plate was transferred into 5 mL of LB broth and incubated at 37 °C for 3–4 h. Following incubation, to determine bacterial suspensions optical density (OD) was assessed at 600 nm using UV–visible spectroscopy and adjusted to a turbidity of 0.5 McFarland, equivalent to 1.5 × 10^8^ Colony forming unit (CFU)/mL (Patel 2017). Then, 100 µL of this suspension was eventually spread on an MHA plate. A nonwoven specimen of 1 × 1 cm was placed on agar, followed by 24 h of incubation at 37 °C in the incubator. At the end of the incubation period, a clear zone around the fabric was measured using a scale that indicates the antibacterial activity. Three replicants were used to measure the zone of inhibition, and the average values were reported. Non-leaching agents did not create an inhibition zone as they did not spread through the agar. The size of the clear zone gives insight into the antimicrobial activity potential and diffusion rate of antimicrobial agents.

The second antibacterial assessment method has been developed for a rapid and simple screening of various kinds of samples and is based on bioluminescent bacteria (Jyske et al. 2023). In this method, the sample nonwoven materials with antibacterial agents were introduced to a genetically modified *E. coli* K12 + pcGLS11 strain. The strain emits a luminescent light signal as a part of its normal metabolism, 4.2 × 10^8^ CFU/mL corresponds to approximately 10^9^ photons/s/cm^2^/sr (sr = steradian, unit of solid angle) luminescent production, and the introduction of antibacterial agents results in a dose-dependent signal reduction. The bacteria in 50% agar content, were poured on the 1 × 1 cm sample sheets placed on solid nutrient agar plates as illustrated in Fig. 1b. Plates were then incubated at 30 °C overnight and scanned using a SPECTRAL Lago X in vivo imaging system. Antibacterial efficiency was calculated as a percentage of inhibition (Tienaho et al. 2015), with standard error and coefficient of variation also determined from triplicate data.

The colony counting quantitative assessment of antibacterial tests was conducted using the absorption method following the ISO 20743:2013 standard which synchronized with the JISL 1902 test method (Pinho et al. 2011). This method is performed for textiles treated with non-leaching biocidal agents under dynamic conditions with the bacterial inoculum directly adsorbed onto fabric samples. An *E. coli* colony was cultured in LB broth and diluted to a concentration of 1 to 3 × 10^5^ CFU/mL following the standard. Treated and reference samples were tested without sterilization to mimic activity in real-life conditions, and the bacterial suspension was inoculated onto a fabric sample of 1 × 1 cm in a tube (Erdogan 2020). Experiments were done with initial microbial concentration determined at time zero and after 24 h of incubation at 37 °C as shown in Fig. 1c. The plate count method was used to assess bacterial colony growth, involving inoculation of a suspension onto nutrient media in a petri plate and counting colonies after incubation. Each sample was replicated three times.

##### ii. Surface charge measurements

To conduct zeta potential measurements, a fabric sample weighing 0.5 g was grinded into small pieces or splinters using a Wiley mill. The particle size was separated using a 60 Å mesh sieve. The grinded sample was soaked in deionized water overnight at room temperature. The Zetasizer Nano-ZS90 (Malvern) was used for zeta potential measurement. The sensor tip was rinsed with deionized water and the instrument was calibrated using a standard sample (− 40 mV ± 5.8 mV) with a particle size of 300 nm. Measurements were performed using the dip cell technique at pH 7.0. Zeta potential readings for the samples were recorded after calibration. Three readings were taken for each sample and the results were averaged.

##### iii. Fabric structural parameters

On the one hand, to understand the amount of chitosan loaded onto the nonwoven and on the other hand, to examine how the basic nonwoven fabric parameters were affected by chitosan treatment, the thickness and weight gain before and after treatment were measured. The thickness of the sample was assessed by employing Lorentzen and Wettre SE250D thickness tester according to the ISO5084 test method. The ten sample specimens were measured and the average values were reported.

The weight gain is calculated by measuring the areal density (AD in g/m^2^) before and after treatment (Gebeyehu et al. 2024) as expressed in Eq. 1.

1 $$Weight\,gain \,\left(\%\right)=\frac{Final\,AD-Base\,AD}{Base\,AD} \times 100$$

The sample weight and thickness were measured after being conditioned for 24 h at standard temperature and relative humidity. Three replicants were used to measure the weight of the sample and the average values were reported.

##### iv. Surface characteristics

To assess the binding and chitosan deposition on the surface after post-treatment, the specimens before and after treatment were analyzed by scanning electron microscopy (SEM, Zeiss Sigma VP) and the attenuated total reflection Fourier transform infrared (ATR-FTIR) spectroscopy (PerkinElmer, USA). For SEM measurement the specimen was first coated using 80 Au/20 Pd for about 40 s at 20 mA using a Q150R sputter coater (Quorum Technologies Ltd.) to enhance electron emission. The surface of the sample is scanned through a focused beam of electrons to produce an image and the sample images were taken by a secondary electron detector with an acceleration voltage of 1.3–3 keV.

ATR-FTIR spectroscopy was used to determine the surface functional groups in cellulosic nonwoven after chitosan coating based on characteristic peaks. The FTIR spectra were recorded by a standard optical system with KBr windows and a Lithium tantalate (LiTaO_3_) detector in a spectral range of 4000–500 cm^−1^ by mean of 16 scans at a resolution of 4 cm^−1^ with a signal-to-noise ratio (SNR) of 9.300:1.

##### v. Bending resistance and bending stiffness

The bending resistance and stiffness were measured using the L&W bending tester as per ISO 2493:1992 standards. The instrument was calibrated as per the recommendation of the manufacturer and the bending length was set to 10 mm, and the desired bending angle was selected to be either 7.5 degrees or 15 degrees (SCANDINAVIAN 1995). Rectangular specimens (38 × 17 mm) were conditioned at standard temperature and humidity before testing. During testing, one end of the sample was clamped, and the surface of the test piece was placed in contact with a blunt knife edge mounted on a force sensor. Up on the testing, the instrument bends the sample to the predetermined angle, and the force required to achieve this bend was measured. This force is a direct indication of the bending properties of the material. Measurements were conducted on five samples, and the mean value was reported.

##### vi. Mechanical properties

The tensile strength at tensile strain and modulus of the pristine and treated specimen was tested in accordance with ISO 9073–18 test standards employing a Universal Instron 4204 tensile strength tester. Initially, the samples were prepared with a specimen preparation template in the machine direction. Then, the fabric was extended at a 50 mm/min extension rate using a constant rate of loading extension of 1 kN. Any breaks that occur near the jaws or at loads substantially less than the average that gave outliers were rejected. As a result, an average of a minimum of five samples were reported.

##### vii. Thermal properties

Thermogravimetric analysis (TGA) was conducted using a TGA 5500 instrument to evaluate the thermal stability of nonwoven samples before and after treatment with chitosan. The treated nonwoven material was first cut into smaller pieces and dried in an oven at 80 °C to remove moisture. For each analysis, approximately 5–10 mg of the dried sample was weighed and placed into a platinum pan. The temperature was programmed to increase from 30 °C to 800 °C at a rate of 20 °C per minute. Throughout the analysis, the weight loss of the sample was continuously monitored to assess its degradation behavior. The changes in the onset temperature of degradation, the peak degradation temperature and the final residue at 800 °C were used to compare the thermal stability of treated and untreated samples.

## Results and discussion

The nonwoven fabric was post-treated using three different molecular weights, four different concentrations, and two post-treatment techniques with a chitosan solution as the antimicrobial agent. Both physio-mechanical properties and antibacterial assessments were successfully conducted to evaluate changes by the treatment. Optimization of the antibacterial properties was carried out for all four concentrations as illustrated in Figs. 5, 6, 7, 8 and 9. However, physio-mechanical analysis was only performed for the concentrations that exhibited a significant change in antimicrobial properties.

### Surface properties

#### Surface morphology

**Fig. 2:**
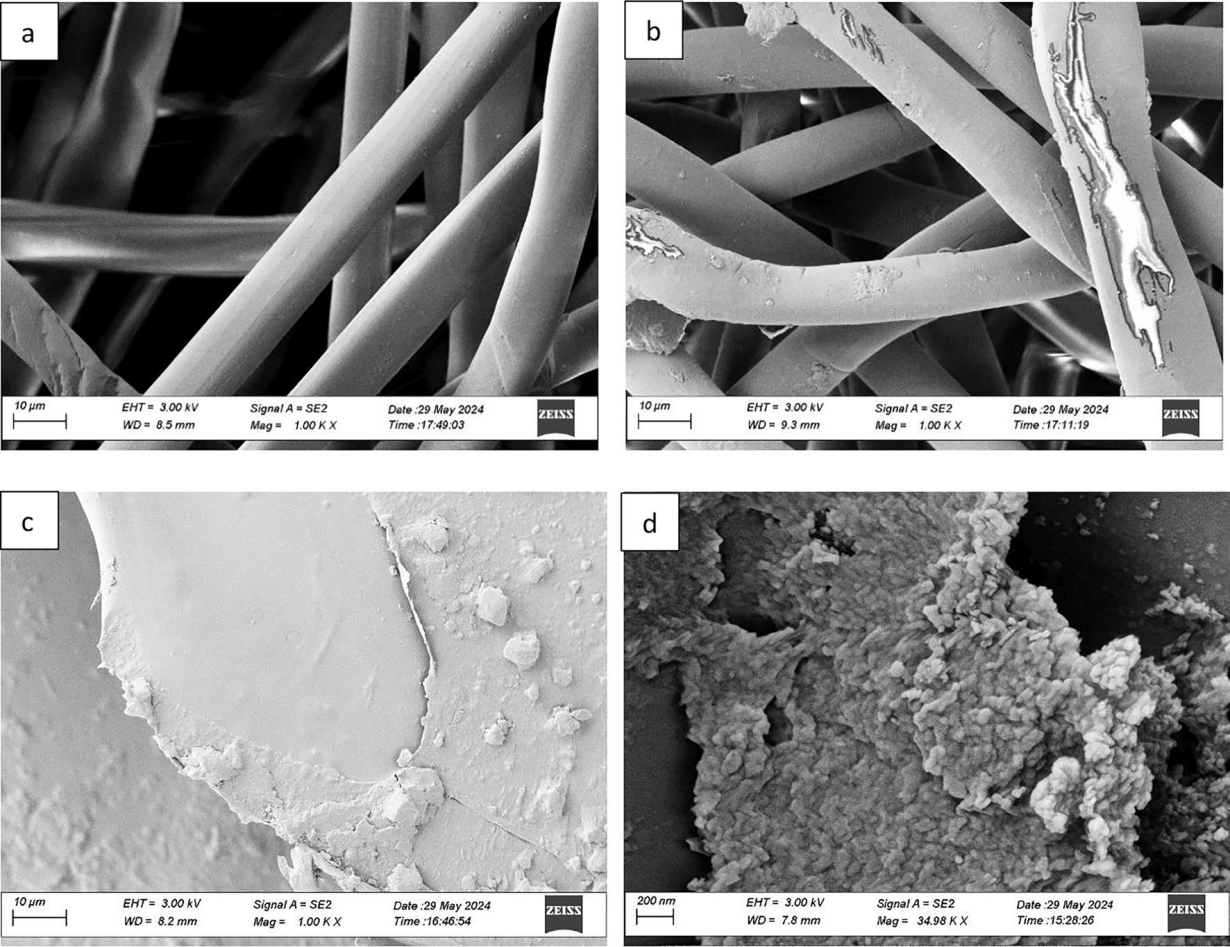
SEM images of **a** Reference nonwoven (R) and low molecular weight chitosan coated with **b** 5 g/L P × D and (C) 5 g/L **d**10 g/L D × D samples

The deposition of chitosan on the nonwoven surface was confirmed by SEM micrographs as shown in Fig. 2. The untreated nonwoven fabric in Fig. 2a had a smooth surface. In contrast, the pad-dry sample in Fig. 2b showed sparse chitosan deposition on the surface. A similar result was observed in the SEM image of pure cotton and chitosan padded fibers (Zhou and Kan 2014). Whereas in the dip-dry sample, the formation of a chitosan layer was observed. This difference is likely due to the nipping pressure during the padding process which squeezed out excess chitosan or pushed it inside the fibers, leaving less on the surface as also observed in weight gain measurements in Fig. 4. The dip-dry method avoids this pressure, allowing for greater retention and forming a thicker surface coating.

The adherence of chitosan onto cellulosic fibers occurred due to the presence of a similar backbone between chitosan and cellulose and associated molecular interaction as demonstrated in previous studies (Laine et al. 2002; Zhou and Kan 2014).

Chitosan concentration also affected the surface texture. At the initial concentration of 5 g/L shown in Fig. 2c, the coating was smooth and even compared to 10 g/L (Fig. 2d) where the deposition appeared denser. Higher concentrations caused chitosan to clump together, forming a thicker layer and enhanced surface coverage. This increased deposition may provide barrier points for microorganisms possibly improve the antibacterial properties of the material. Overall, this distinction highlights deposition efficiency varies by application method, molecular weight and concentration which potentially may affect antibacterial performance which is a function of chitosan deposited on cellulose.

#### Surface chemistry

**Fig. 3:**
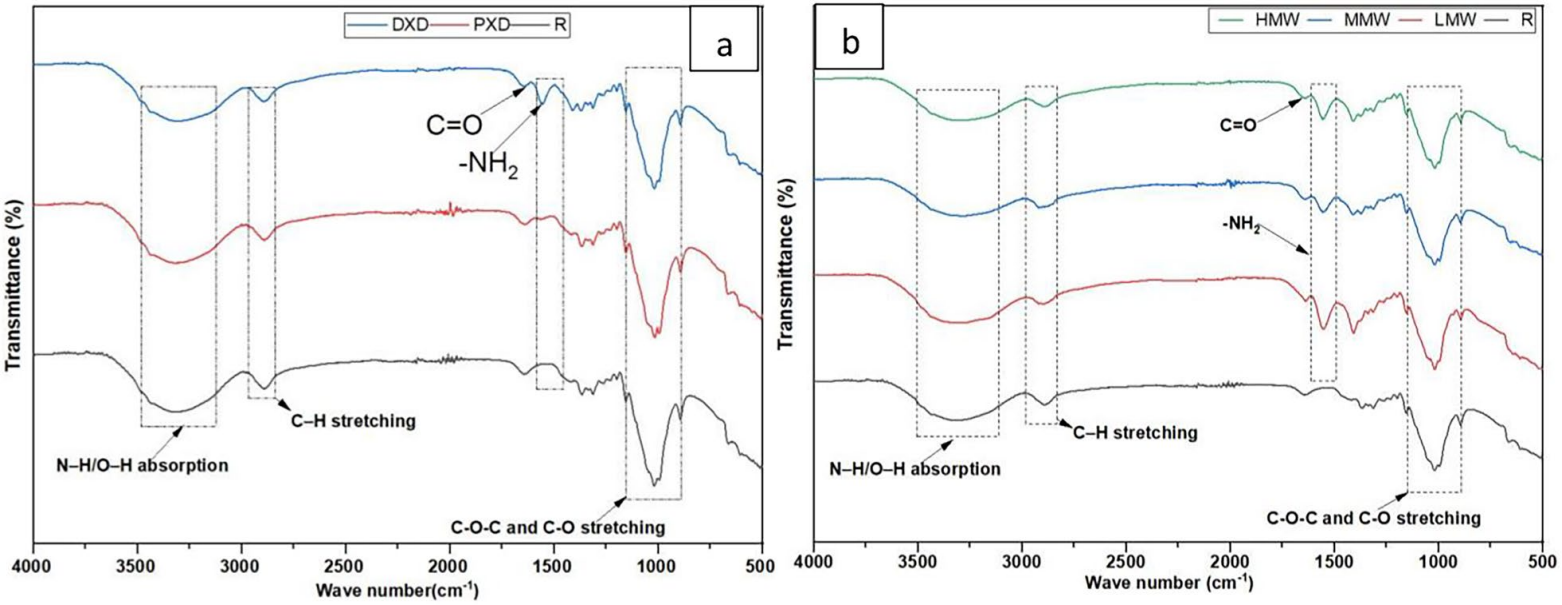
FTIR spectra of chitosan-coated nonwoven in **a** different application methods for LMW 5 g/L and **b** D × D of different molecular weights

FTIR analysis can provide detailed information about the chemical interactions and structural changes in cellulose treated with chitosan. As shown in Fig. 3, the broad absorption band observed in the range of 3500–3100 cm^−1^ corresponds to the combined stretching absorption of N–H and O–H groups in treated cellulose. It serves as an indication of hydrogen bonding interactions between cellulosic nonwoven and chitosan showing successful intermolecular interaction of chitosan onto the nonwoven as also revealed by a similar study (Ferrero et al. 2015). The presence of chitosan is also confirmed by a characteristic peak observed exclusively in the treated samples at the peaks 1645 and 1550 cm^−1^ which are attributed to C = O stretching and N–H bending vibrations, respectively, corresponding to the amine groups of chitosan (Pathirana et al. 2023; Sionkowska et al. 2004). The reduced intensity of this peak in LMW 5 g/L pad-dry treated samples suggests fewer amino groups were retained on the surface, likely because the squeezing process during treatment impregnated molecules into the fibers or removed excess material.

The interaction between chitosan and cellulosic nonwoven is also driven by electrostatic interactions and van der Waals forces in addition to hydrogen bonding. The absorption peak in the 1600–1370 cm⁻^1^ region represents free amino groups on the surface. These amino groups were protonated (-NH₃⁺) in acidic solutions that could enable electrostatic binding with the negatively charged hydroxyl groups of cellulose (Jabli et al. 2011). Another peak at 1150–1050 cm⁻^1^ assigned to C–O–C or C–O stretching reflects structural compatibility between chitosan and cellulose further supporting the effective deposition of chitosan onto the nonwoven fabric. The polysaccharide backbones of chitosan and cellulose share similar structural features, as highlighted in prior studies (Nishiyama et al. 2002; Pratama et al. 2019). This structural similarity not only reinforces the stability of the chitosan coating but also enhances its functional properties by promoting strong molecular interactions. These hydrogen and ionic bonds indicate a stable chitosan coating on cellulosic nonwoven.

### Structural properties of fabric: weight gain and thickness

**Fig. 4:**
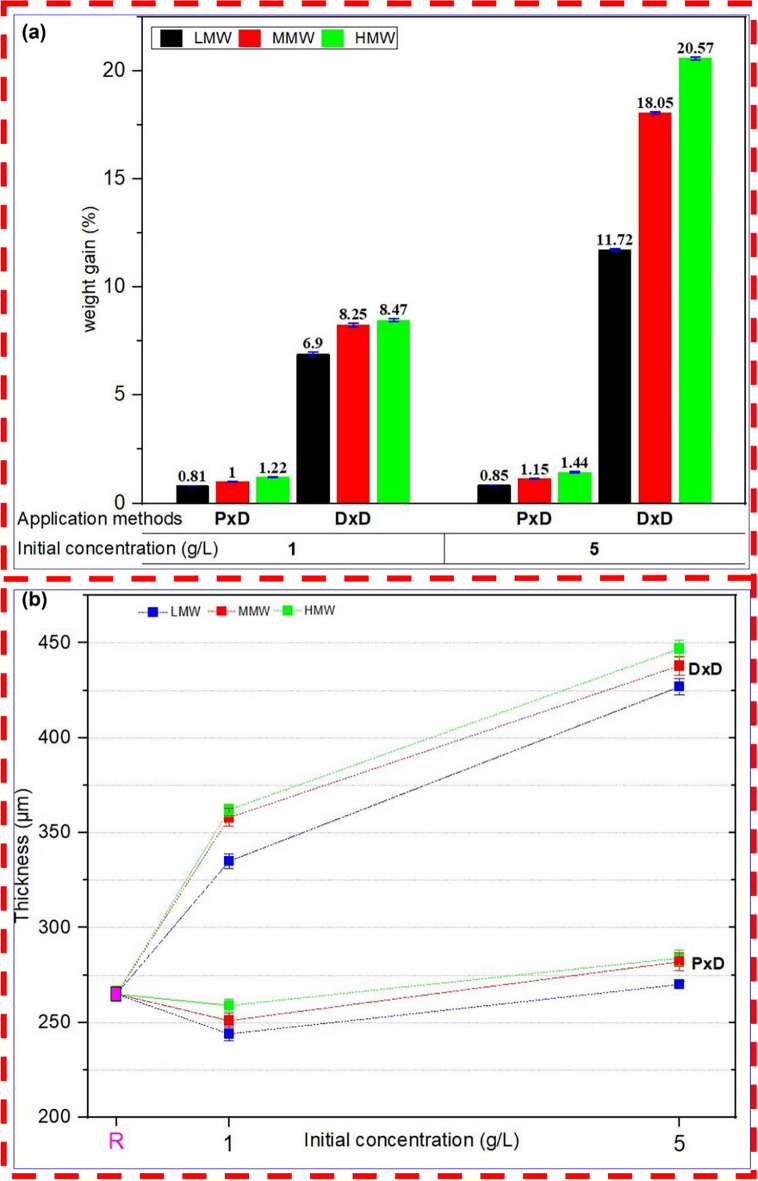
Effect of molecular weight, concentration and application method on basic structural parameters **a** weight gain of treated samples **b** thickness for reference sample (R) and treated samples

The application method, concentration and molecular weight also affect weight gain and thickness as shown in Fig. 4. Despite the identical initial concentration of chitosan in the solution, the actual amount deposited onto cellulose differed significantly by these factors. The changes in weight by treatment are shown in Fig. 4a. While application methods are considered, the pad-dry application method resulted in a lower weight gain compared to dip-dry coating. This is because the pressure in the nipping of the padding process removes excess chitosan solution compared to the dipping process. The weight gain increases as the concentration and molecular weight of chitosan increases. Higher concentrations of chitosan provide more material for deposition, while higher molecular weights mean larger chitosan molecules, both contributing to greater weight gain (Ibrahim et al. 2016). Previous studies also reported an increase in the weight of the cellulosic fabric when they are crosslinked with chitosan and cyanuric chloride (Sadeghi-Kiakhani et al. 2018).

In post-treatment of antibacterial finishing, thickness, and weight are closely correlated, indicating that as the weight gain increases, the thickness also correspondingly increases. The thickness and weight of the chitosan-treated specimen were preserved in the LMW chitosan-coated cotton sample in the pad dry method. Yet, the small change in weight gain % and thickness shows that the chitosan in both application methods is loaded onto the textiles. The weight and thickness of nonwoven fabrics are crucial factors in determining their suitability for various applications. These parameters significantly influence the performance characteristics of materials, such as absorbency, strength, durability and comfort (Albrecht et al. 2006). For example, in healthcare settings such as wound dressings, surgical drapes, and incontinence pads thicker and bulkier nonwovens are preferred for their superior absorbency and durability for managing bodily fluids (Amiot et al. 2014; Dutkiewicz 2002). Conversely, the lighter and thinner fabrics provide comfort and flexibility. In personal care products, for instance, baby wipes, facial cleansing wipes, and feminine hygiene pads, the weight and thickness of the nonwoven may be tailored to provide comfort and flexibility (Shirvan and Nouri 2020).

The relationship between structural changes and their practical application for specific functions remains a topic for further study. However, as a general rule of thumb, fabric parameters should be designed in the fabric manufacturing and maintained after finishing treatments. Therefore, an appropriate selection of treatment methods and proper optimization of parameters of chitosan solution is necessary to maintain weight and thickness in nonwoven fabrics to ensure functional performance and user satisfaction for a specific application.

### Antibacterial properties

Antibacterial agents used in textile treatments can be leaching or non-leaching agents depending on diffusion properties and mechanism of action. The textile surface containing leaching antimicrobial agents slowly releases the biocide agents acting as venom to slowly kill its surrounding microbes. These agents diffuse to a certain area establishing a zone of inhibition (Shahidi and Wiener 2012). The non-leaching compounds, adhered strongly to the surface of the treated textile surface either with ionic bonds or covalent bonds, do not discharge into the surrounding environment. They show their activity only when they come in contact with microorganisms. Upon contact, the molecules interfere with the bilipid layer of the bacterial cell membrane, readily moving into cells, altering its metabolic activity, and leading to bacterial death (Sawan et al. 1998).

In some papers and industrial reports, the antimicrobial properties of textiles are reported arbitrarily, using both absorption and diffusion methods, regardless of whether the antimicrobial agent is leaching or non-leaching. This approach can lead to misleading conclusions. For example, diffusion test methods may yield inaccurate results when evaluating non-leaching agents, as these agents rely on direct microbial contact rather than diffusion into the surrounding environment. The correct way to report these properties is to carefully align the test method with the mechanism of action of the antimicrobial agent. In this paper, three types of test methods were used to evaluate the antimicrobial properties of chitosan-treated cellulosic nonwoven materials, and the results for each were distinct and consistent.

Chitosan shows antibacterial effects by neutralizing the negative charges present on the surfaces of bacteria. The amine groups in chitosan interact with the bacterial cell wall to kill bacteria or inhibit their growth. This phenomenon can be analyzed using zeta potential (Chandrasekaran et al. 2020) which measures the surface charge shifts caused by the deposition of free amino groups of chitosan onto cellulosic nonwoven. Table 2 illustrates the zeta potential of pristine and treated samples. The untreated cellulosic nonwoven exhibited a zeta potential of − 26.6 mV. After treatment with chitosan, the surface negativity decreased due to the shifting of the isoelectric point to a higher pH region. This is obvious since protonated chitosan provides free amino groups, which eventually reduce the negativity of the cellulose surface (Strnad et al. 2010). Besides, chitosan adsorbed on the surface makes the surface compact and this leads to a reduction of the hydration of the fiber interior. Thus, shifting the negative values of the untreated samples towards positive values for the treated samples. Similar results were also obtained in the work carried out in the previous research (Sadeghi-Kiakhani et al. 2018).

**Table 2:** Surface charge for pristine and chitosan-coated nonwovens

Sample code	Sample description			Zeta potential (mV)
	Application method	Molecular weight	Concentration (g/L)	
Reference (R)	Controlled sample			− 26.6$\pm 0.8$
L1	D × D	LMW	1	14.3$\pm 0.7$
L2	D × D	LMW	5	24.9$\pm 0.3$
L3	D × D	LMW	10	17.1$\pm 0.7$
L4	D × D	LMW	15	24.4$\pm 0.3$
M1	D × D	MMW	1	20.5$\pm 0.4$
M2	D × D	MMW	5	25.4$\pm 0.9$
M3	D × D	MMW	10	37.6$\pm 0.7$
M4	D × D	MMW	15	32.3$\pm 0.5$
H1	D × D	HMW	1	15.9$\pm 0.5$
H2	D × D	HMW	5	28.0$\pm 0.6$
H3	D × D	HMW	10	33.6$\pm 0.5$
H4	D × D	HMW	15	18.2$\pm 1.2$
L5	P × D	LMW	1	− 15.4$\pm 0.8$
L6	P × D	LMW	5	− 12.5$\pm 0.7$
L7	P × D	LMW	10	− 06.8$\pm 1.7$
L8	P × D	LMW	15	00.9$\pm 0.5$
M5	P × D	MMW	1	− 12.8$\pm 1.4$
M6	P × D	MMW	5	− 06.1$\pm 0.7$
M7	P × D	MMW	10	− 00.1$\pm 0.2$
M8	P × D	MMW	15	05.8$\pm 0.1$
H5	P × D	HMW	1	− 15.7$\pm 1.0$
H6	P × D	HMW	5	− 03.1$\pm 0.4$
H7	P × D	HMW	10	08.9$\pm 0.8$
H8	P × D	HMW	15	12.2$\pm 1.7$

The order of antibacterial activity corresponded to the zeta potential of chitosan-treated nonwovens (Chandrasekaran et al. 2020; Du et al. 2009). Dip-coating resulted in higher zeta potential values than pad-coating due to greater chitosan deposition. Increasing chitosan concentration and molecular weight further elevated zeta potential, directly enhancing antibacterial activity. This is attributed to the increased availability of positively charged amino groups, which interact with negatively charged bacterial surfaces, disrupting their structure and leading to bacterial death. As shown in Table S1, samples with higher zeta potential exhibited stronger bacterial elimination. This highlights the need to optimize chitosan treatment conditions and application methods to achieve higher zeta potential values and desired antibacterial properties in cellulosic nonwovens.

#### Antibacterial activity based on agar diffusion test

Agar diffusion test was employed in the qualitative assessment of chitosan-treated cellulosic nonwovens for their antibacterial effectiveness. Figure S1 showed that both pristine and treated cellulosic nonwovens did not exhibit any inhibition of bacteria growth near laid samples, suggesting that chitosan does not leach when attached to cellulosic nonwoven. Even though the SEM results confirmed the presence of chitosan molecules on the surface of the cellulosic nonwoven material, the lack of bacterial inhibition may be attributed to the strong binding of chitosan to the loose and porous structure of the cellulosic nonwoven. This binding is facilitated by hydrogen bonding, hydrophobic interactions and electrostatic interactions justified with ATR-FTIR analysis (Fig. 3). The strong intermolecular interaction makes it unlikely for chitosan to leach into the surrounding environment. Consequently, chitosan remains firmly attached to the cellulosic nonwoven, preventing it from creating any inhibitory zones in the agar diffusion test (Dhiman and Chakraborty 2015).

Many researchers have confirmed that chitosan is a non-leaching antimicrobial (Erdogan 2020; Gzyra-Jagieła et al. 2016; Ibrahim et al. 2021; Rinaudo 2006). Although, there are some papers showing that chitosan is a leaching agent with an inhibition zone in agar diffusion test (Chandrasekar et al. 2014; El-Tahlawy et al. 2005; Zhou and Kan 2014). This should be clear that there is a discrepancy in the literature. Hence, we are confirming here that our findings support the first hypothesis about this. The clear region can be observed only if the agents are loosely bound with the fabric sample and diffuse through agar leaving from the treated samples otherwise the agents will not be able to show any zone of inhibition. However, only performing agar diffusion tests does not confirm the antimicrobial efficacy of agents. In addition, the presence of a zone of inhibition in a sample does not necessarily show that it kills microorganisms. It might act as a bacteriostatic agent with the ability to inhibit the growth and multiplication of bacteria (Ristić et al. 2011).

Despite the agar diffusion test being an easy and fast test method, it provides very unreliable results for non-leaching antibacterial agents such as chitosan. Thus, the antibacterial efficacy of non-leaching agents like chitosan-treated cellulosic non-woven should be evaluated by using different standardized test methods (Pinho et al. 2011).

#### Antibacterial activity assessment using luminescent biosensor method

In addition to the diffusion test, a rapid luminescence-based method was employed. It is based on the recombinant indicator bacteria, which show a rapid luminescent light reduction directly proportional to the decrease in metabolic activity when in contact with antibacterial substances (Vesterlund et al. 2004). The approach differs from the diffusion method as the bacteria is poured on top of the sample fabrics. In our study, we tested chosen samples using this method to measure the percentage of bacterial inhibition against gram-negative *E. coli* strain. Sample 2, as shown in Figure S2, displayed a 3.7% inhibition rate, which was not considered significant, whereas all other samples tested negative for test with *E. coli* bacteria indicating that the metabolic activity of the strain is increased as seen in Figures S2 and S3. The outcome observed for *E. coli* closely resembled the results of the agar diffusion test.

None of the current non-woven samples with chitosan coating indicated any activity against the strain although it was observed in Fig. 3 that chitosan was attached to cellulosic fibers (Strnad et al. 2010). Previously, it has been found that high molecular weight chitosan shows activity against the strain both as itself and in woven textiles with chitosan microencapsulated plant extract treatments (Kunnas et al. 2024). It is possible that the method would require higher concentration of the effective substrate as whole well region of interest is used. Another reason could be that chitosan is known to possess bactericidal activity by binding to bacterial cell membranes, which can interfere with cell permeability and cause leakage of intracellular components (Li et al. 2010; Liu et al. 2004). This effect is often contact-based and might not be as effective in a gel-like medium such as 50% agar where the bacteria are not in immediate proximity to the chitosan surface. Additionally, although the luminescent signal used as an indicator of metabolic activity is highly sensitive it may not reflect slow or low-level antibacterial effects that occur over an extended period. Hence, if the interaction between the chitosan-treated material and the bacteria is weak or gradual, it may not lead to an immediate and substantial drop in luminescence, particularly in short-duration experiments when using less aggressive antibacterial agents like chitosan against the more resistant gram-negative bacteria. We have previously suggested that as low as 20% inhibition is significant (Kunnas et al. 2024). Thus, this test was not found to be optimal for evaluating the antibacterial efficacy of the current chitosan-treated cellulosic nonwovens.

#### Quantitative antimicrobial test based on colony counting

The preliminary screening of chitosan-treated samples via agar diffusion test did not provide any support to show that chitosan-treated cellulosic non-woven are leaching antibacterial agents. Although none of the samples exhibited diffusible activity, studies showed that chitosan is an excellent antimicrobial compound that can show antibacterial activity upon direct contact with bacterial suspension (Strnad et al. 2010). Hence, the absorption test which is very accurate and sensitive test method (Pinho et al. 2011) for non-leaching agents were performed. In this method, the bacterial cells directly adhere to nonwoven surfaces, which provides quantitative results for antibacterial efficiency. The bacterial efficiency was evaluated based on log reduction (log_10_) of viable bacterial concentration in treated samples in comparison to the control sample after a certain incubation time.

The number of bacterial cells in all the test specimens was calculated using the colony count method. Table S1 showed the survival number of bacteria recovered from the specimens at time zero and 24 h incubation. The number of *E. coli* recovered upon immediate contact with untreated nonwoven was equal to 1.72 × 10^5^ ± 1.13 × 10^4^ CFU/mL while after 24 h of incubation, the number of bacteria recovered from untreated non-woven was found to be increased to 3.02 × 10^6^ ± 8.33 × 10^5^ CFU/mL. A maximum number of bacteria were recovered from cellulosic non-woven upon elution with 0.85% saline solution. Bacteria adhere very loosely onto the negative surface charge of cellulosic nonwoven resulting in maximum recovery of bacteria upon elution. In addition, the increase in the number of bacteria upon 24 h of incubation showed that the cellulosic nonwoven favors the growth of bacteria under favorable environmental conditions. Cellulosic fibers become an excellent medium for the growth of microorganisms under moist and warm temperatures (Montazer and Afjeh 2007).

**Fig. 5:**
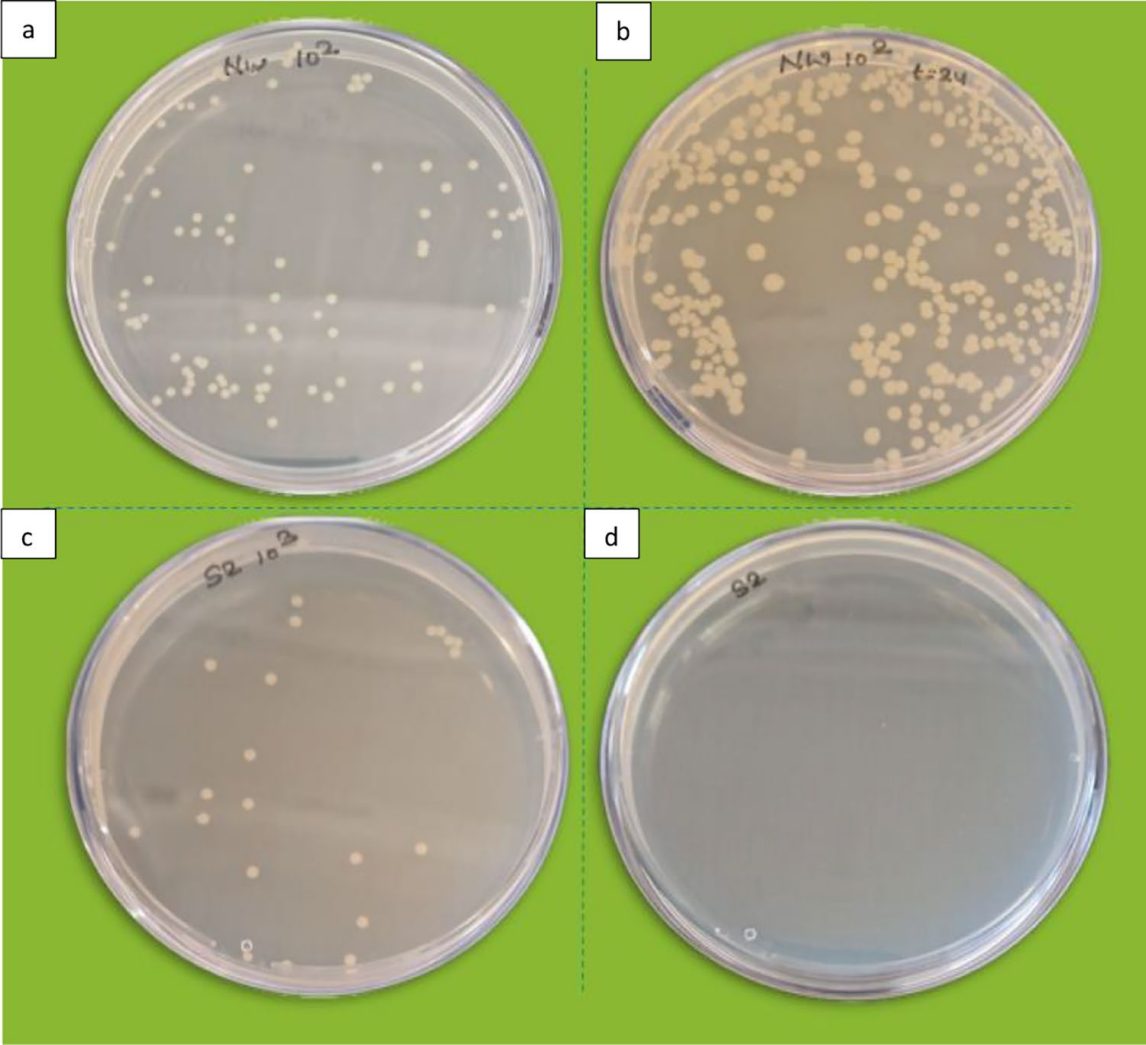
Viable bacterial colonies recovered from sample R at **a** T = 0 h and **b** T = 24 h and from L6 at **c** T = 0 h and **d** T = 24 h

Among all treated specimens, samples L6, M6, and H5 and all dip-dry samples treated with 1,5, 10, and 15 g/L showed 100% lytic activity against *E. coli*. The number of bacteria recovered immediately after inoculation of *E. coli* on sample L6 at zero time was 6.2 × 10^4^ ± 2.4 × 10^4^ CFU/mL and for sample L1 was 1.64 × 10^5^ ± 5.09 × 10^4^ CFU/mL. Upon elution of these samples after 24 h of incubation, no bacterial colonies were observed. Figure 5 illustrates bacterial colonies recovered from untreated nonwoven and sample L6 at T = 0 h and T = 24 h to demonstrate antibacterial activity. Thus, L6 was considered as the minimum concentration for the treatment of the nonwoven. The reason for the death of test bacteria was due to the attribution of the protonated amino group of chitosan which eventually interacts with an anionic surface of *E. coli* (El-Tahlawy et al. 2005). The damage to the outer membrane of *E. coli* caused by the interaction of chitosan even at its lowest concentration of 0.1% was observed through transmission electron microscopy in another study (Li et al. 2010). The bacterial count was found to be reduced by tenfold in pad-dry samples except for samples L6, M5, and H5 while a significant reduction in bacteria was observed for all dip-dry samples by 10–30-fold except for low molecular dip-coated samples of L2, L3, and L4 at time zero compared to control sample.

The reduction in many bacterial colonies in the treated sample in comparison to the control sample showed that upon immediate contact, bacteria with negative surface charge adsorb onto the surface of cellulosic fabric very quickly. The rod-shaped cellular structure of the bacteria is also one of the factors that provide a large surface area for adhesion onto chitosan-treated cellulosic fibers. The bacteria attached very firmly to the positively charged chitosan-cellulosic surface due to strong electrostatic interaction between positive and negative charge (El-Tahlawy et al. 2005). Thus, the study supports our result that due to more adherence of bacteria with an increase in the concentration of chitosan, a smaller number of bacteria were eluted from chitosan-treated-cellulosic nonwovens. The elution of bacteria from the fabric after 24 h of incubation showed no colonies from samples treated with 5, 10, and 15 g/L concentrations of LMW, MMW, and HMW chitosan-treated samples by both pad-dry and dip-dry processes. Shin and Min (Li et al. 2010) reported that polypropylene nonwoven fabric treated with chitosan of molecular weight of 100 kDa showed effective inhibition against *E. coli* at 1% treatment concentration and with 210 kDa at 0.3%.

The pad-dry process was found to be less effective for 1 g/L both LMW and MMW-treated non-wovens. These two specimens showed less biocidal activity. While all dip-dry samples showed effective biocidal activity against test bacteria. No colonies were obtained for all dip-coated samples upon elution after 24 h of incubation. The effective biocidal activities were observed because of the strong adhesion of bacterial cells onto chitosan-coated nonwovens (Egorov et al. 2023). In comparison to pad-dry samples, dip-coated samples showed a high reduction in test bacterial load. This is because, in pad-dry samples, much smaller grain-size chitosan molecules have adhered onto the cellulosic fibers compared to dip-coated samples as seen in SEM images Fig. 2. The chitosan solution formed a film and showed its maximum deposition on treated fabrics. It showed that due to the high density of amino groups present in dip-coated samples (Table 2) than in pad-dry, a greater reduction in the number of recovery bacteria was observed in dip-coated samples than in pad-dry ones.

**Fig. 6:**
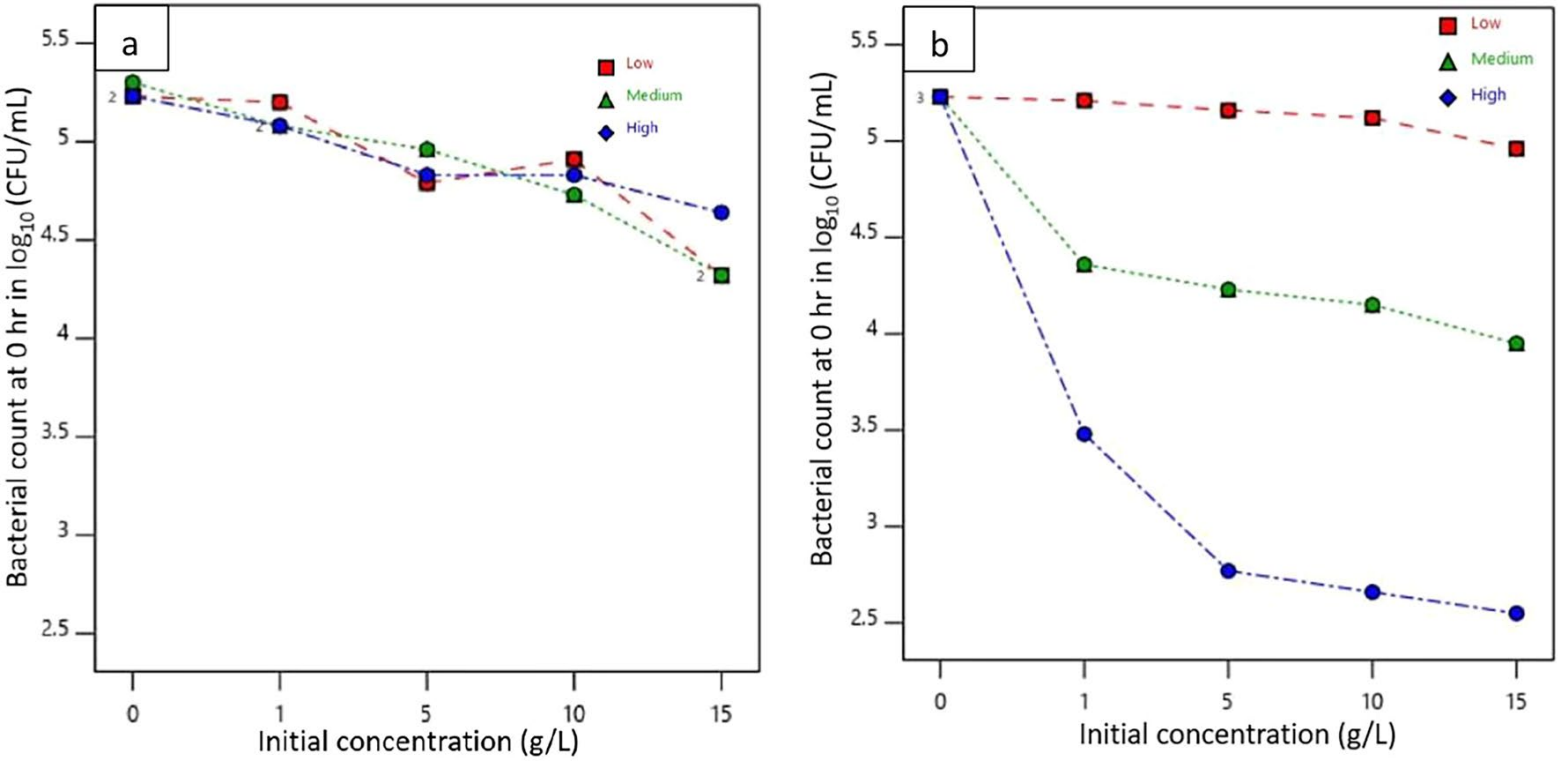
Interaction effect of concentration and molecular weight on bacterial count reduction at initial interaction (0 h) in **a** padding and **b** dipping application

The log reduction of bacteria upon the interaction of concentration and molecular weight of chitosan in both padding and dipping applications is graphically represented in Fig. 6. It was analyzed by using a split-plot factorial design via Design-Expert (DX11) software. The graph of pad-dry samples at initial bacterial interaction showed that for the same concentration of different molecular weights of chitosan, log reduction values did not vary significantly. It indicates despite different molecular weights if the concentration of chitosan is the same then all pad-dry fabrics will show similar interaction against *E. coli*. However, a significant difference in log reduction of test bacteria was observed with differences in molecular weight and concentration for dip-dry specimens. A remarkable difference in log reduction was found in the dip-coated HMW chitosan-treated sample than in MMW and LMW chitosan-treated nonwovens. Upon treatment with different concentrations of HMW chitosan solution by dip-coating method, the log value was reduced to around 3 for the concentration of 1 g/L and then further gradually decreased up to 2.54 with an increase in the concentration to 15 g/L comparison to the log value of the control sample which was equal to 5.24.

**Fig. 7:**
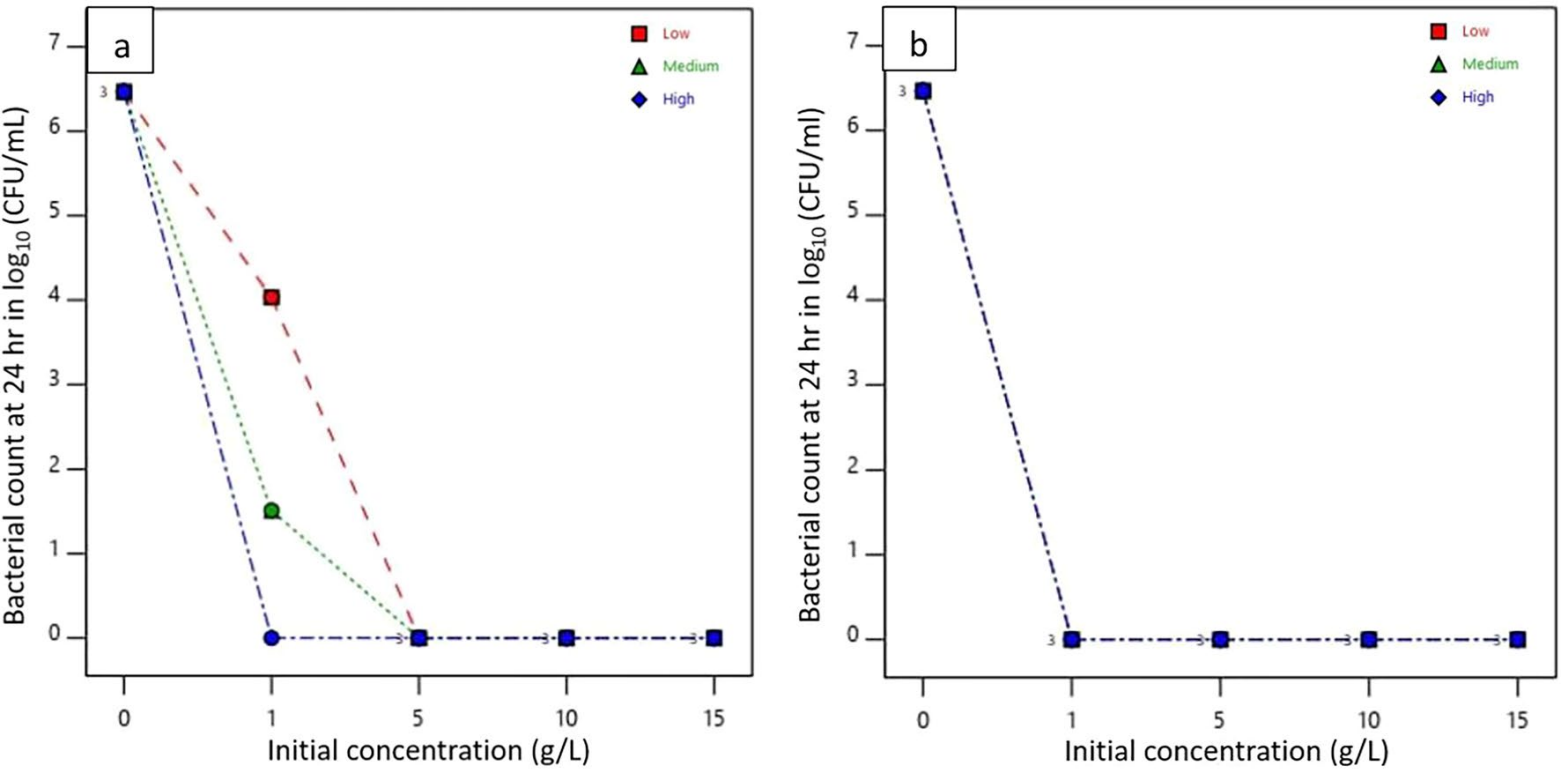
Interaction effect of concentration and molecular weight on bacterial reduction after 24 h. in padding (**a**) and dipping (**b**) application

The reduction in bacterial count of treated samples after 24 h of incubation is shown in Fig. 7. Except for samples L5 and M5, all treated samples reduced bacterial counts to zero from an initial log count of 6.48 in the control sample. The bacterial reduction for L5 and M5 nonwovens was calculated to be 4.03 and 1.51, respectively. For dip-coated samples, all treated samples showed complete bacterial reduction with no recovery, unlike the control samples.

All bacteria were removed from nonwoven surfaces during the initial interaction, as indicated in Fig. 6. Therefore, the results after 24 h of incubation suggest that the interaction between chitosan and bacterial cells was responsible for killing nearly all viable bacteria immobilized on the nonwoven surfaces during incubation at an optimal temperature. Table 3 also provides information that with a 95% confidence level, there is a significant difference in antibacterial properties of treated nonwoven among different molecular weights, application methods and their combined effect on the antimicrobial activity of chitosan-treated samples at the initial interaction. Though the bacterial count reduces in all molecular weights and application methods, for an efficient reduction at initial interaction a higher molecular weight of chitosan through dipping application as shown in Fig. 6 performs best with a model desirability of 0.882. A significant interaction at initial concentration is important as it prevents the spread and multiplication of pathogens in application areas where there is a high risk of contamination.

**Table 3:** Effects of weight, application method, and their interaction effect on the antimicrobial activity of chitosan-treated samples at initial interaction

Source	Degree of freedom	F-Value	p-Value	Decision
Model	5	14.32	< 0.0001	Significant
Molecular weight	2	12.28	0.0003	Significant
Application method	1	18.34	0.0004	Significant
Interaction effect	2	13.89	0.0002	Significant

**Fig. 8:**
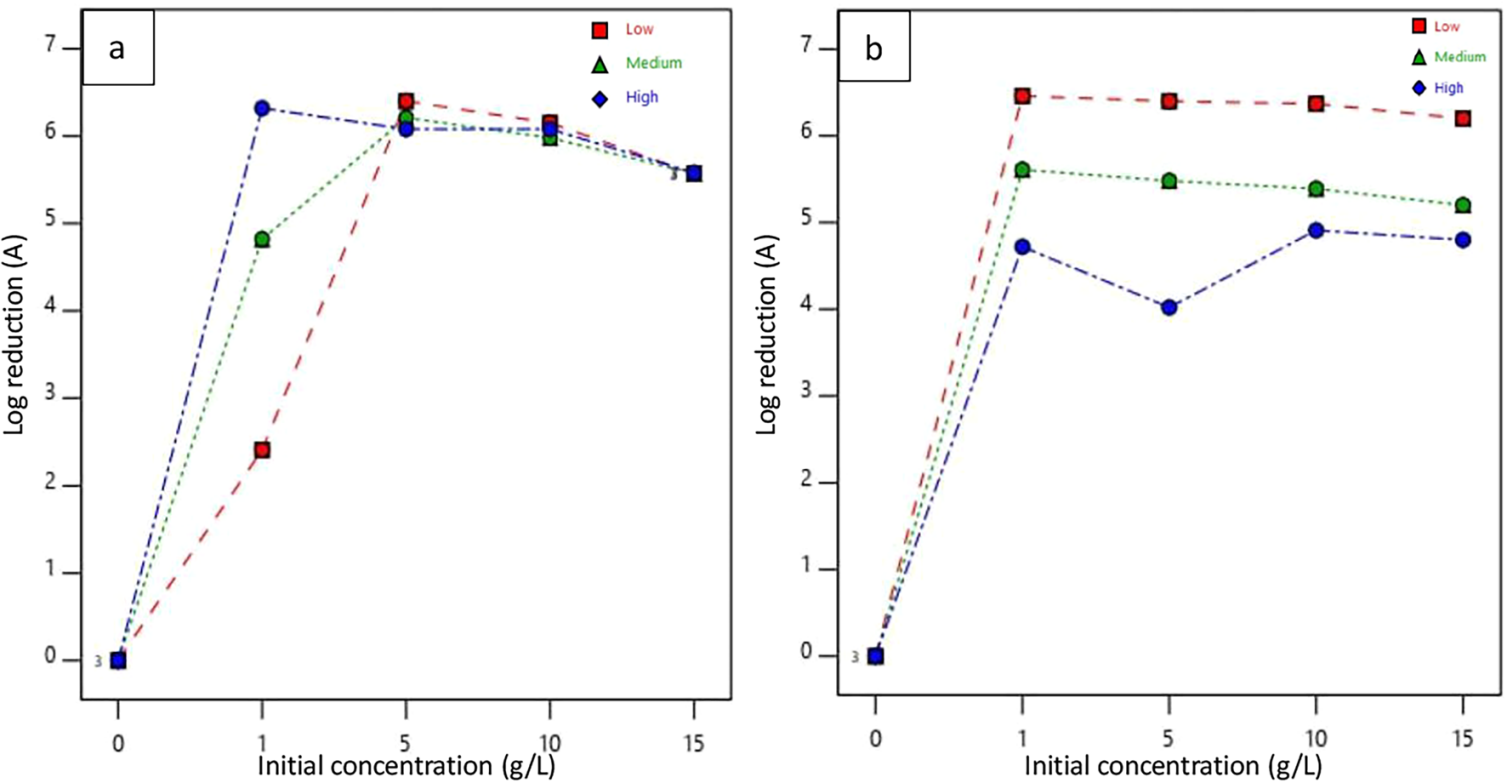
Interaction effect of concentration and molecular weight on log reduction in padding (**a**) and dipping (**b**) application

Antibacterial activity was calculated in terms of log reduction from bacterial count and comparative effect of concentration and molecular weight of chitosan along with two application processes are presented in Fig. 8. The maximum antibacterial activity (A) was calculated in samples H5 which were equal to 6.32 and 6.46 respectively. According to the JIS L 1902 test method, the test specimen is considered to have a good antibacterial effect if its antibacterial activity value is greater than 3 (99.9% bacterial reduction) and if its value is 2 $\le$ A < 3, then it is considered to exhibit some effect against the growth and multiplication of bacteria. Among all padded and dip-coated samples, only L5 showed an antibacterial activity equal to 2.41. Hence, for LMW a concentration of chitosan equal to 1 g/L can be considered as bacteriostatic concentration for samples prepared by padding application. This value exhibits that it can inhibit the growth and multiplication of bacteria but cannot kill it. In the padded sample, the variation in antibacterial activity was observed with only LMW, MMW, and HMW chitosan at a concentration of 1 g/L. However, except for sample L1, the antibacterial activity value was measured above 3. With an increase in concentration from 5, 10, and 15 g/L, the antibacterial activity values were observed to reach a saturated point that is between 5 and 6 for all three molecular weight chitosan.

In the case of the dip-coated sample, all 1, 5, 10, and 15 g/L of LMW chitosan showed high activity compared to nonwoven treated with HMW chitosan. This is because low molecular weight chitosan can easily penetrate thinner cell membranes of gram-negative bacteria due to its smaller particle size in comparison to high molecular weight (Fu et al. 2011). HMW chitosan forms a coating on the cell wall of gram-negative bacteria, which causes inhibition in the transport of nutrients into bacterial cells (Zheng and Zhu 2003). Thus, LMW chitosan-treated nonwoven showed antibacterial activity due to its ability to interfere with cytoplasmic components, alter metabolic pathways, and arrest protein cascade while HMW chitosan-treated nonwoven kills bacteria via nutrient deprivation. However, all dip-coated samples showed a measurement of antibacterial activity value greater than 3, signifying that all dip-dry samples showed good antibacterial activity against test bacteria *E. coli*. It was reported in previous research work that (Dann and Hontela 2011) the activity of chitosan varies depending on its molecular weight. The study showed that antibacterial activity was increased with an increase in molecular weight from 5000 to 9.18 × 10^4^ while varying molecular weight in between 9.16 × 10^4^ to 1.08 × 10^6^, it showed reduced antibacterial activity.

**Fig. 9:**
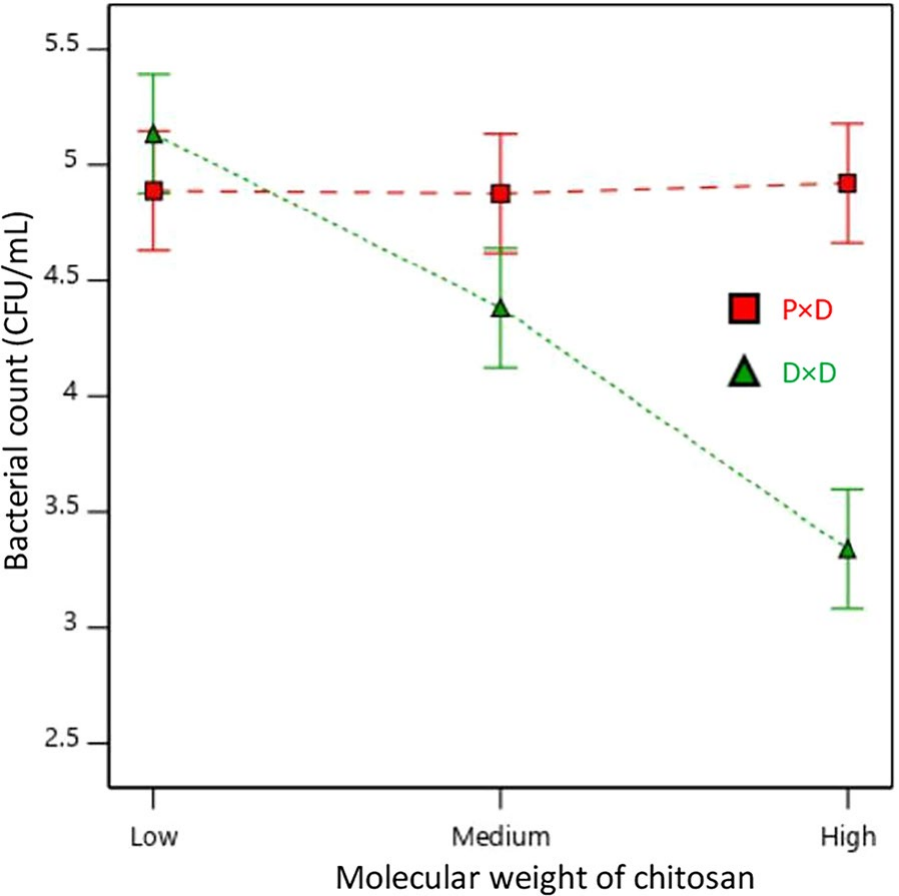
Interaction effect of padding and dipping application method in bacterial reduction (CFU/mL) corresponding to molecular weight

The combined effect of effect of application method and molecular weight in bacterial reduction is summarized in Fig. 9. A significant difference of bacterial reduction in pad-dry and dip-dry application methods for nonwoven treated with different molecular weight of chitosan was observed. The bacterial reduction was consistent between low, medium, and high molecular weight chitosan, while a significant variation in bacterial reduction in terms of CFU/mL can be observed for dip-dry nonwoven samples. LMW dip-dry samples showed a maximum reduction in bacterial colonies in test samples than in HMW dip-dry test samples.

The antibacterial activity of chitosan-treated cellulosic nonwoven generally shows higher antibacterial activity compared with the antibacterial activity of chitosan in the culture medium shown in Table 4 especially at lower concentration levels. This better performance can be attributed to the way chitosan is restrained on the fabric surface, allowing for more direct and sustained contact with bacteria. In contrast, chitosan in the culture medium is dispersed, leading to less direct interaction with bacterial cells.

**Table 4:** Antimicrobial properties of chitosan in culture medium

Concentration of chitosan (g/L)	Antibacterial activity		
	LMW	MMW	HMW
1	3.79	6.14	5.04
5	6.14	7.78	7.63
10	7.49	6.69	7.29
15	7.73	7.26	6.51

Additionally, the structure of nonwoven fabrics helps trap bacteria on the surface, making them more vulnerable to the antibacterial effects of chitosan. This better performance of nonwoven-treated chitosan has significant benefits. The restrained chitosan provides sustained antibacterial action, making it more effective in applications where continuous bacterial control is needed, such as medical textiles, hygiene products, and filtration systems. Moreover, the targeted use of chitosan in nonwoven fabrics ensures efficient antibacterial protection while reducing waste, making it a practical and sustainable solution for real-world applications.

### Bending resistance and bending stiffness

The resistance of fabric to bending or fabric stiffness is used to understand the flexural rigidity, bending modulus, and bending length of a nonwoven textile. These parameters are vital for determining material performance across various applications (Moghassem 2012) like medical textiles, geotextiles, automotive textiles, and hygiene products. Nonwoven fabrics are utilized in healthcare applications for surgical gowns and masks, where appropriate stiffness ensures user comfort and ease of handling. Geotextiles depend on stiffness for soil stabilization and erosion control, while automotive textiles benefit from stiffness in interior linings and insulation. Hygiene products like diapers need optimal stiffness for functionality and comfort. Thus, the measurement of bending resistance and bending stiffness helps manufacturers in improving product comfort, functionality, and durability (Russell 2022).

**Fig. 10:**
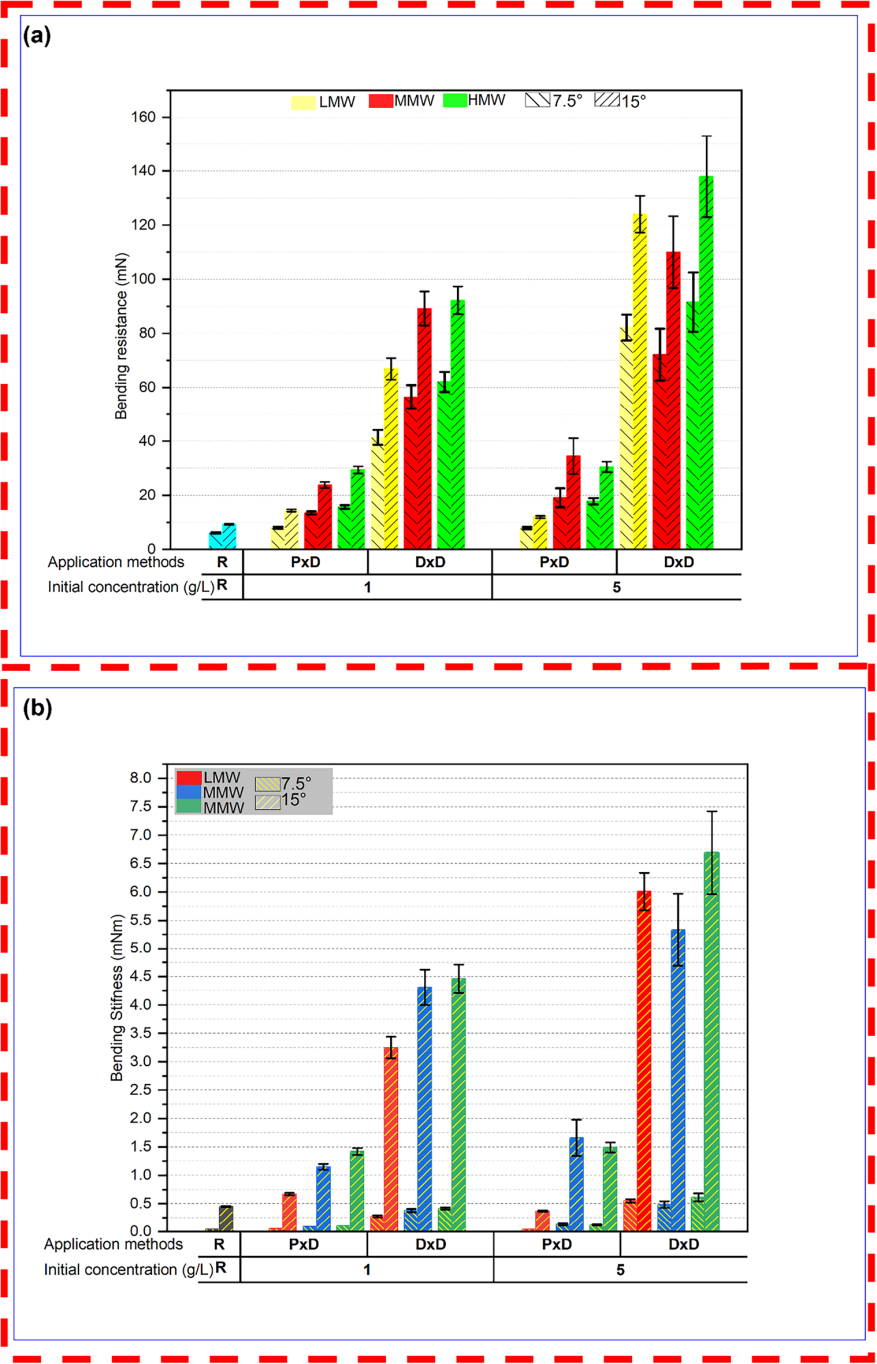
Bending behaviors of controlled and treated samples **a** bending resistance **b** bending stiffness

Figure 10a and b show the bending resistance and bending stiffness of controlled and coated samples respectively. From the general trends of results, it can be seen that the higher concentrations across all molecular weights and higher molecular weight chitosan-treated cellulosic nonwoven resulted in greater bending resistance and bending stiffness (Lee et al. 1999). This can be attributed to the increased stiffness and mechanical strength imparted by the chitosan, as higher concentrations and molecular weight form a more coverage and film network within the fiber matrix (Strnad et al. 2010). The dip-dry application makes the fabric stiffer and makes it rigid compared to the padding method, likely due to more deposition of chitosan in the dipping application which reinforces the nonwoven structure more effectively (Strnad and Zemljič 2023). As the molecular weight increases, the length of polymer chains in chitosan increases which might create more entanglements and stronger intermolecular interactions with cellulose fibers, leading to a higher rigidity. The bending resistance and bending stiffness at 15° angle were generally higher than at 7.5°. At small angles, the response might be predominantly elastic, but as the angle increases, the material can transition into a different deformation regime that exhibits higher resistance to further bending indicating that a higher angle may result in stiffness and resistance.

### Mechanical properties

To analyze the mechanical properties of the nonwoven before and after chitosan treatment, a specimen of different samples was subjected to an axial load until failure and the breaking load and extension were recorded. It is a measure of durability, safety, integrity, and functionality for different application areas (Rawal et al. 2010).

**Fig. 11:**
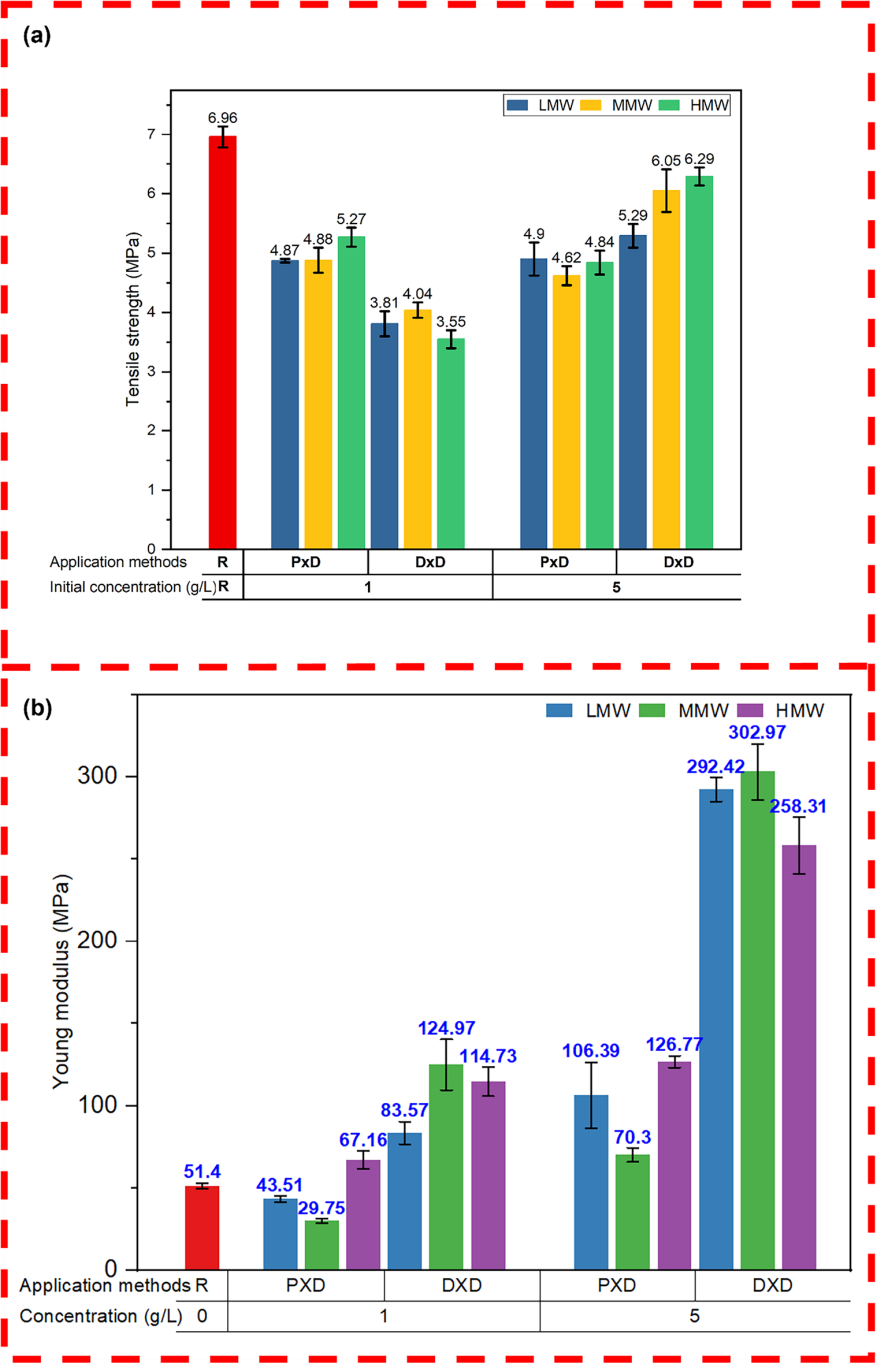
Mechanical properties of different specimens **a** tensile strength at tensile stress **b** Young modulus

Figure 11a illustrates the tensile strength of nonwoven materials treated with chitosan at different concentrations and application methods. The reference sample had a tensile strength of 6.96 MPa, which decreased after treatment. A general decrease in tensile strength may be associated with increased stiffness after treatment as shown in Fig. 11b which again resulted in the reduction of the sliding part of each fiber that may reduce the uniform distribution of the exerted mechanical forces on the substrate and or the acidic condition (Cheng et al. 2014) may slightly affect the integrity of cellulose. At 1% concentration, the padding method was more effective in maintaining the tensile strength of materials, possibly due to a more uniform application and better surface coating of chitosan. Higher chitosan concentrations and molecular weights (Gupta and Haile 2007) in the dipping method yielded tensile strength results comparable to the reference sample (Arain et al. 2013). The dipping method at higher concentrations may be attributed to a layer of chitosan coating onto the nonwoven matrix, leading to better eternal reinforcement and improved mechanical properties. Increasing the molecular weight also generally shows higher strength, which may be associated with better reinforcement due to stronger networks within the material.

Figure 11b shows the Youngs modulus of samples pristine and coated samples. Generally, a more deposition of chitosan on the surface of cellulosic nonwoven makes it more stiff and increases Youngs modulus. This is mostly because as a result of the chitosan coating, the degree of freedom of fibers inside the structure may be reduced due to the film deposition on the surface of the fiber which in turn increases the stiffness of the nonwoven (Jeong and Bae 2009). Higher molecular weight polymers tend to stay on the fiber's surface because they cannot pass through the pores in cotton fiber due to the relatively small pores found in the cell wall of cellulosic fiber. Here, Medium molecular weight chitosan showed the highest young modulus compared to low and high molecular weight chitosan. Since the solution is slightly viscous for HMW chitosan, there might be a possibility of uneven deposition of chitosan and thus results in less young modulus compared to MMW chitosan.

### Thermal properties

**Fig. 12:**
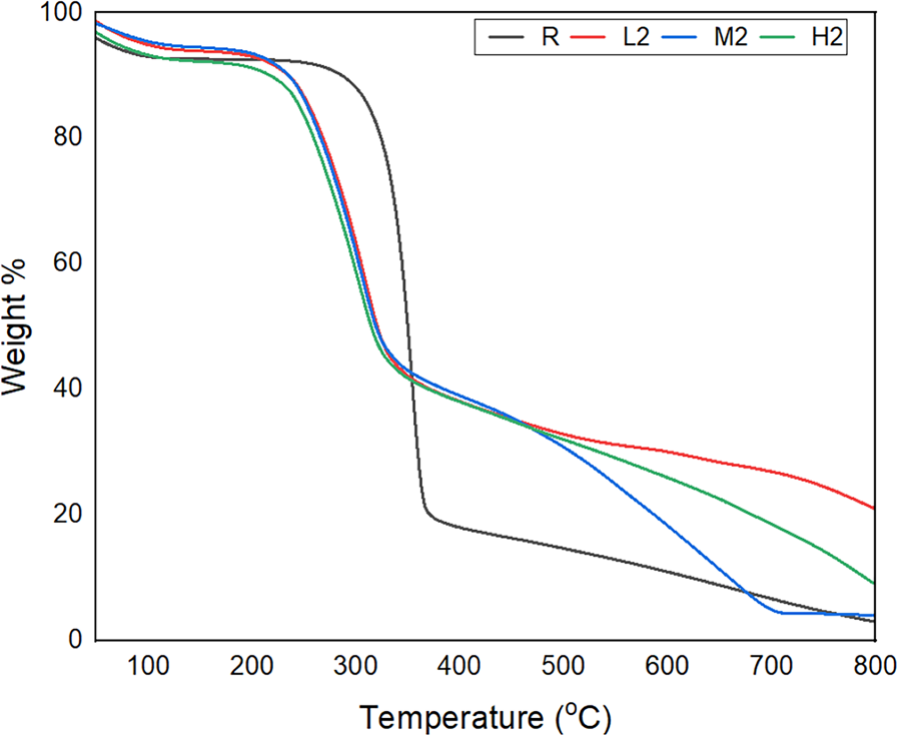
Thermal analysis of reference and selected treated samples

For application areas like decoration and even causal apparel, thermal stability or the gradual decomposition of textiles is necessary as it prevents quick failure by flame or fire. Even though the aim of the study was not to enhance thermal stability; we investigated how the nonwoven behaves before and after chitosan treatment. The pristine nonwoven has higher initial thermal stability, starting its decomposition at 330 °C than all the chitosan-coated samples as depicted in Fig. 12. The onset degradation temperature of chitosan was reported at 254 °C in the previous studies which are aligned with this study for the treated samples (Arora et al. 2011). However, once decomposition begins, it proceeds rapidly with a high rate of weight loss (R_max_ of—0.9511%/°C), resulting in significant weight reduction (76.84%) over a narrow temperature range. In contrast, treated samples L2, M2, and H2 begin to decompose at significantly lower temperatures but exhibit a more gradual weight loss. As the molecular weight increases, the weight losses are lower for all coated samples and the rate of decomposition is much slower with in an increase in the molecular weight as shown in Table 5. This indicates that the chitosan coating may provide a thermal stabilization layer that could slow down its degradation process (Mosaad et al. 2022). This may be explained by the coating of chitosan on cellulosic nonwoven fibers introduces additional binding that promotes thermal resistance. The lower initial decomposition temperatures of the chitosan-coated samples can be attributed to the decomposition of the chitosan itself, which starts earlier than the cellulose fibers. However, the presence of chitosan delays the complete degradation of the cellulose, resulting in a more gradual weight loss and lower residual weight at elevated temperatures.

**Table 5:** Major thermal indices of treated and untreated samples

Samples	Initial degradation Temp	Final degradation Temp	% of decomposition	Weight loss	R_max_ (%/^0^C)
R	330	364	76.84	3.923	−0.9511
L2	256	332	58.75	3.943	−0.2579
M2	251	326	59.68	3.135	−0.3034
H2	247	327	64.59	3.977	−0.2385

## Conclusion

The antimicrobial finishing of cellulosic nonwovens is increasing because of its significance for concerns of microbial growth, pathogenic disease transmission, and the need for sustainable, biodegradable alternatives to synthetic materials. This study investigates the effects of chitosan on the intrinsic and antimicrobial properties of cellulosic nonwovens and the mechanism of action. The findings illustrate the role of molecular weight, concentration and application method in influencing both the antibacterial and physicomechanical properties of the treated nonwovens.

Dip-dry application was shown to result in greater chitosan deposition on the nonwoven compared to the pad-dry method, a finding corroborated by SEM analysis and this change affects the performance of treated nonwovens. The results of colony counting test revealed that chitosan proved to be a non-leaching antibacterial agent, requiring direct contact for antibacterial assessment. The antibacterial mechanism against *E. coli* was attributed to the cationic amino groups and molecular size of the chitosan, with smaller molecules facilitating quick penetration into the bacterial cells. In terms of mechanical properties, chitosan-treated samples exhibited increased bending resistance and stiffness, although tensile strength decreased. Thermal analysis further showed that chitosan coatings provide a stabilizing effect by slowing the thermal degradation of the nonwovens. Based on the findings, chitosan-treated nonwovens hold a promise potential for single use application in areas such as medical, hygiene and filtration as indicated by a review research (Pia Hautamäki 2024; Saha and Tehrani 2024).

The antibacterial activity against gram-positive bacteria like *Staphylococcus aureus* was beyond the scope of this study. Future research should aim to test the effectiveness of chitosan against both gram-positive and gram-negative bacteria and molecular and cellular characterization of bacterial death could be explored to establish its broader antimicrobial spectrum. Additionally, further studies could investigate the impact of lower chitosan concentrations, fire resistance, and scalability for industrial applications. These findings highlight the potential of chitosan-treated nonwovens for medical, hygiene, and other protective applications, offering an environmentally friendly alternative to synthetic materials.

## Supplementary Information

Additional file 1.

## Data Availability

The data used to support the findings of this study are included within the article.
