# Supplementary material for: Antibacterial and physicomechanical properties of cellulosic nonwovens functionalized with chitosan: a study on interaction effects of influencing factors and assessment methods
Source: Bioresour Bioprocess. 2025 Feb 15;12(1):11. doi: 10.1186/s40643-025-00843-2 (PMC11829891; doi:10.1186/s40643-025-00843-2)
Supplement: Supplementary file 1 — Additional file 1. [file 40643_2025_843_MOESM1_ESM.docx]

Supporting documents for ‘Antibacterial and physicomechanical properties of cellulosic nonwovens functionalized with chitosan: A study on interaction effects of influencing factors and assessment methods’

Table S1: Bacterial colonies for reference and treated samples

| Sample code | Microbial count (CFU/mL) | |
| --- | --- | --- |
|  | 0 h | 24 h |
| R | 1.7×10^5^ ± 1.1×10^4^ | 3.2×10^6^ ± 8.3×10^5^ |
| L1 | 1.6×10^5^ ± 5.1×10^4^ | 0 |
| L2 | 1.4×10^5^ ± 9.9×10^3^ | 0 |
| L3 | 1.3×10^5^ ± 2.8×10^3^ | 0 |
| L4 | 9.1×10^4^ ± 1.2×10^4^ | 0 |
| M1 | 2.3×10^4^ ± 5.7×10^3^ | 0 |
| M2 | 1.7×10^4^ ± 5.7×10^3^ | 0 |
| M3 | 1.4×10^4^ ± 5.7×10^3^ | 0 |
| M4 | 9.0×10^3^ ± 4.2×10^3^ | 0 |
| H1 | 3.0×10^3^ ± 3.3×10^3^ | 0 |
| H2 | 5.9×10^2^ ± 7.7×10^2^ | 0 |
| H3 | 4.6×10^2^ ± 2.0×10^2^ | 0 |
| H4 | 3.5×10^2^ ± 8.5×10^1^ | 0 |
| L5 | 1.6×10^5^ ± 1.4×10^4^ | 1.1×10^4^ ± 2.7×10^3^ |
| L6 | 6.2×10^4^ ± 2.4×10^4^ | 0 |
| L7 | 8.1×10^4^ ± 9.9×10^3^ | 0 |
| L8 | 2.1×10^4^ ± 2.8×10^3^ | 0 |
| M5 | 1.2×10^5^ ± 5.7×10^3^ | 3.2×10^1^ ± 3.1×10^1^ |
| M6 | 9.2×10^4^ ± 4.2×10^3^ | 0 |
| M7 | 5.4×10^4^ ± 4.2×10^4^ | 0 |
| M8 | 2.1×10^4^ ± 4.4×10^3^ | 0 |
| H5 | 1.2×10^5^ ± 1.4×10^5^ | 0 |
| H6 | 6.8×10^4^ ± 7.8×10^4^ | 0 |
| H7 | 6.8×10^4^ ± 8.1×10^4^ | 0 |
| H8 | 4.4×10^4^ ± 4.9×10^4^ | 0 |


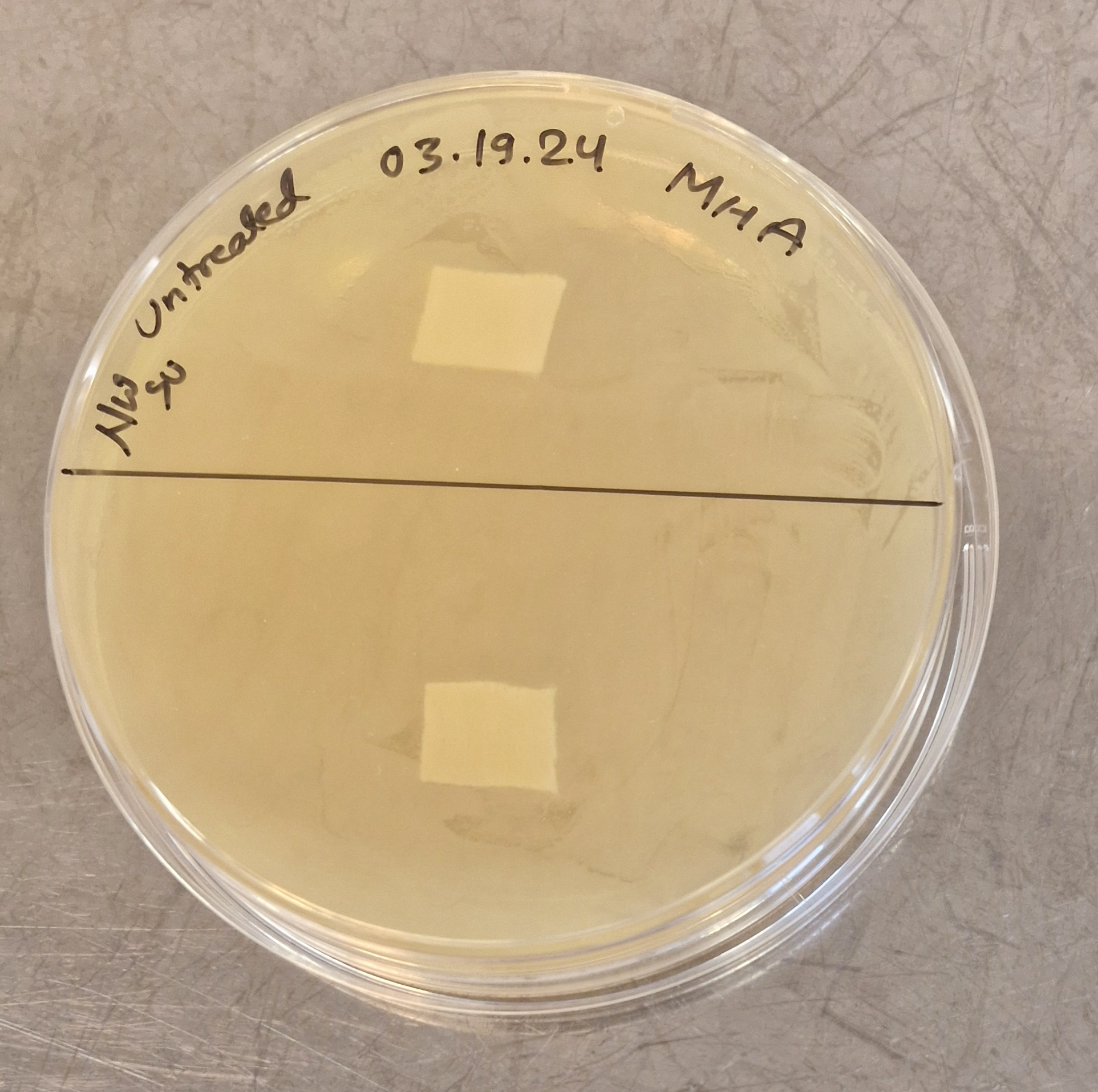

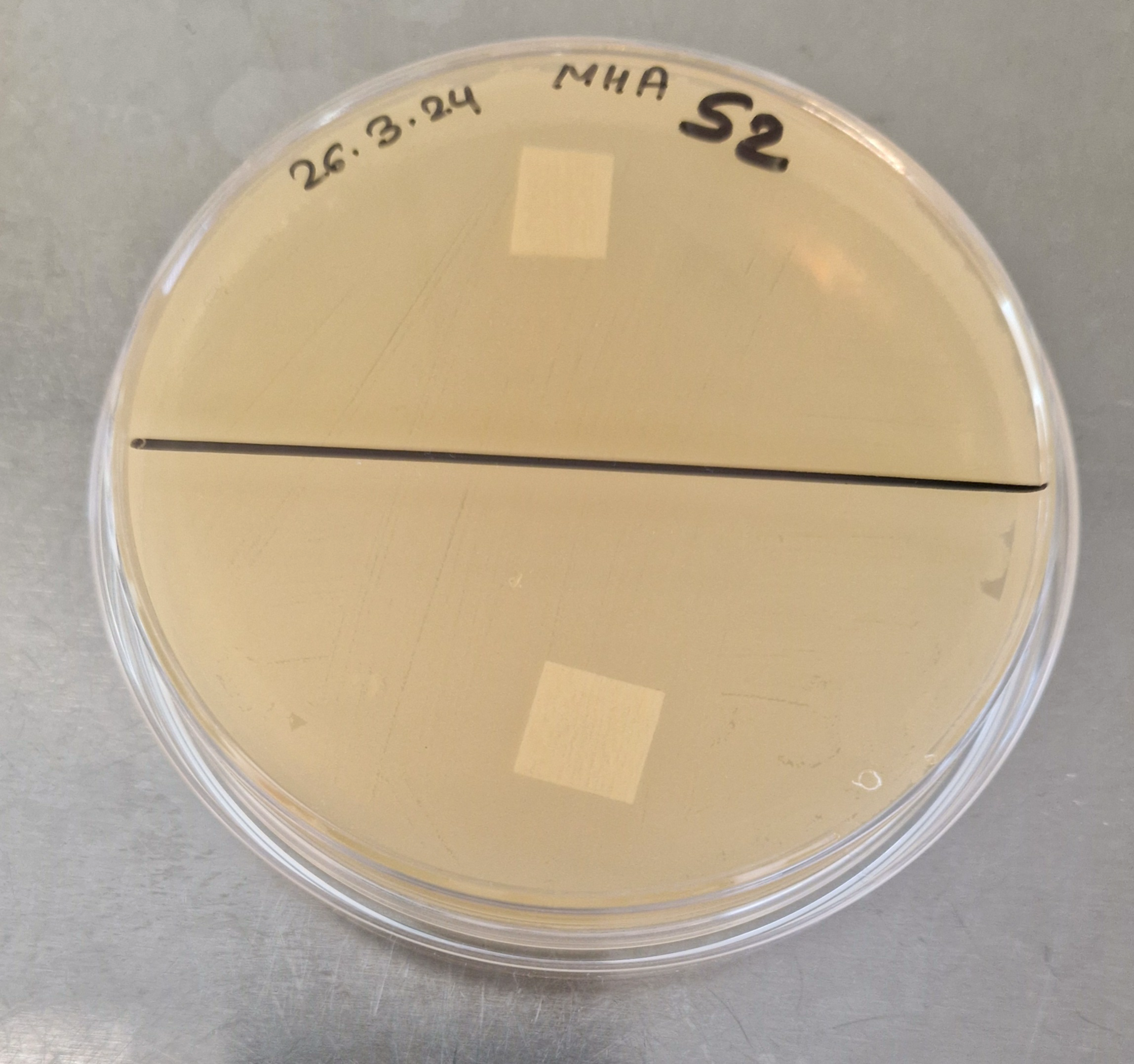


Figure S1: Agar diﬀusion test of untreated nonwoven (left) and LMW 5g/L chitosan-treated nonwoven by pad-dry method (right)





Figure S2: Antimicrobial activity of (Ref) Reference sample, treated samples LMW at (1) 1g/L (2) 5g/L (3) 10g/L and (4) MMW 10 g/L (5) HMW 10 g/L and (6) LMW 15 g/L against the bioluminescent *E. coli* strain. CTRL represents the free growth of the bacterial strain without sample addition (set to 0% inhibition) and 0-CTRL has no bacteria added (set to 100% inhibition). Negative inhibition indicates that the luminescence production is increased instead of decreasing – no antibacterial activity was detected.


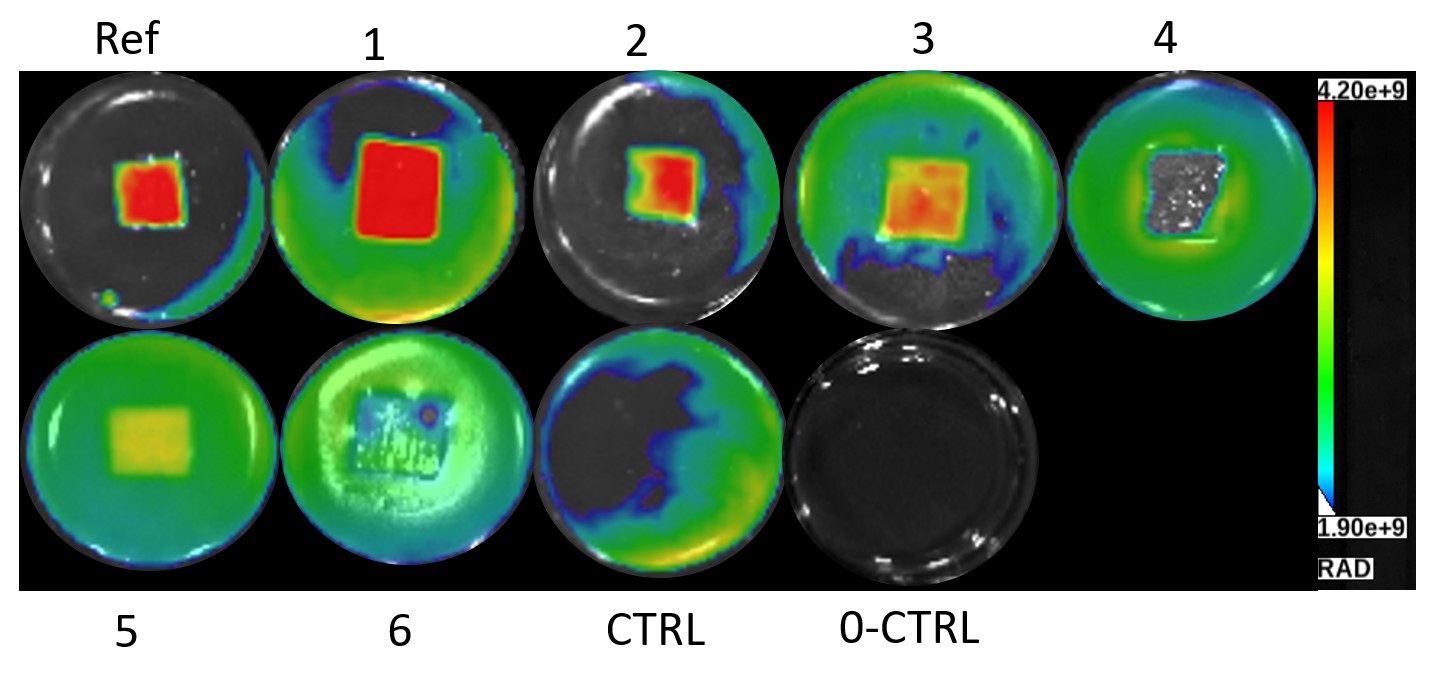


Figure S3: Living bioluminescent bacterial biosensor strain E. coli K12+pcGLS11 with samples (Ref) Reference sample, treated samples LMW at (1) 1g/L (2) 5g/L (3) 10g/L and (4) MMW 10 g/L (5) HMW 10 g/L and (6) LMW 15 g/L. CTRL represents the free growth of the bacterial strain without sample addition and 0-CTRL has no bacteria added. Only examples of the raw data images are shown, while all the samples were plated in triplicates.
